# Cost-effectiveness of Screening Program for Chronic Q Fever, the Netherlands

**DOI:** 10.3201/eid2602.181772

**Published:** 2020-02

**Authors:** Pieter T. de Boer, Marit M.A. de Lange, Cornelia C.H. Wielders, Frederika Dijkstra, Sonja E. van Roeden, Chantal P. Bleeker-Rovers, Jan Jelrik Oosterheert, Peter M. Schneeberger, Wim van der Hoek

**Affiliations:** National Institute for Public Health and the Environment, Bilthoven, the Netherlands (P.T. de Boer, M.M.A. de Lange, C.C.H. Wielders, F. Dijkstra, W. van der Hoek);; University Medical Centre Utrecht, Utrecht, the Netherlands (S.E. van Roeden, J.J. Oosterheert);; Radboud university medical center, Nijmegen, the Netherlands (C.P. Bleeker-Rovers);; Jeroen Bosch Hospital, ’s-Hertogenbosch, the Netherlands (P.M. Schneeberger)

**Keywords:** Q fever, Coxiella burnetii, cost-effectiveness, screening, economic evaluation, the Netherlands, bacteria, zoonoses

## Abstract

In the aftermath of a large Q fever (QF) epidemic in the Netherlands during 2007–2010, new chronic QF (CQF) patients continue to be detected. We developed a health-economic decision model to evaluate the cost-effectiveness of a 1-time screening program for CQF 7 years after the epidemic. The model was parameterized with spatial data on QF notifications for the Netherlands, prevalence data from targeted screening studies, and clinical data from the national QF database. The cost-effectiveness of screening varied substantially among subpopulations and geographic areas. Screening that focused on cardiovascular risk patients in areas with high QF incidence during the epidemic ranged from cost-saving to €31,373 per quality-adjusted life year gained, depending on the method to estimate the prevalence of CQF. The cost per quality-adjusted life year of mass screening of all older adults was €70,000 in the most optimistic scenario.

Chronic Q fever (CQF) is a potentially lethal condition that develops in 2% of Q fever (QF) patients (*1*). QF is caused by infection with *Coxiella burnetii*, a gram-negative bacterium that has its main reservoir in livestock and can infect humans by airborne transmission. CQF can become apparent months to years after infection and usually manifests as endocarditis or vascular infection (*2*). Risk factors for CQF include heart valve disorders, aortic aneurysms, vascular prostheses, older age, and a compromised immune system (*3**–**5*). Prognosis is poor despite long-term antimicrobial drug treatment; 28% of patients need surgery, and 15% die from CQF-related complications (*6*).

During 2007–2010, the Netherlands faced the world’s largest QF epidemic ever documented. More than 4,000 patients with acute QF were notified. However, QF often occurs asymptomatically (*1*), and the total number of infections has been estimated at 50,000 (*7*). Through May 2016, a substantial number of CQF infections occurred, and at least 74 patients died (*8*). Because early detection of CQF might result in a better prognosis, local hospitals initiated multiple targeted screening studies for clinical risk groups living in areas affected by the epidemic. These studies revealed that 7%–20% of screened patients had serologic evidence of *C. burnetii* infection, of whom 5%–31% had CQF (*9**–**11*).

In 2017, new diagnoses of CQF continued to appear in the Netherlands, often with severe complications, and led to a call from multiple concerned parties, including politicians, the QF patient association, and medical doctors for a national CQF screening program. One aspect considered for such a screening program is whether its costs are economically balanced with the expenditure (*12*,*13*). To answer this question, we assessed the cost-effectiveness of a screening program for CQF in the Netherlands.

## Methods

### Overview

**Figure 1:**
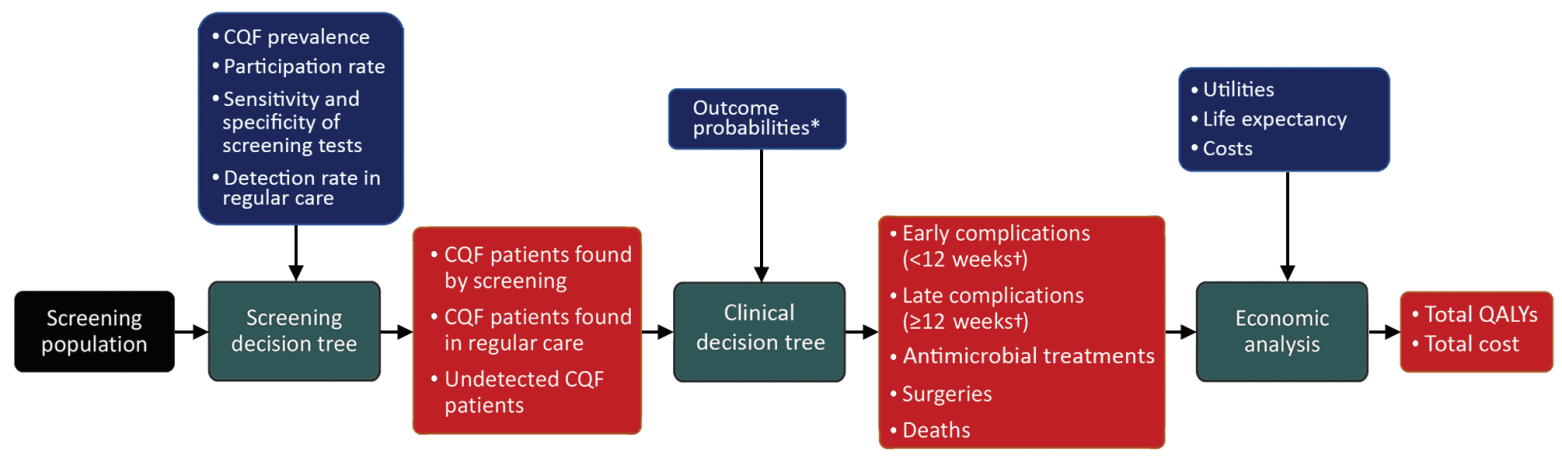
Schematic overview of the health-economic model in a study of the cost-effectiveness of screening for CQF, the Netherlands, 2017. Black square represents model input; green squares are model processes; blue squares are model parameters; and red squares are model outputs. Individual decision trees for screening and clinical outcomes are shown in Appendix Figure 1. *Outcome probabilities differed among patients found by screening, patients found in regular care, and patients who remained undetected. †Weeks after diagnoses. CQF, chronic Q fever; QALY, quality-adjusted life year.

We developed a health-economic decision model to compare estimated costs and effects of a 1-time screening program for CQF with no such screening program (Figure 1). The screening was assumed to occur in 2017, seven years after the epidemic. We estimated comparative outcomes of the model in terms of clinical events, quality-adjusted life years (QALYs), and costs from a societal perspective. We used a lifetime time horizon. Costs were annually discounted at 4% and QALYs at 1.5% (*14*).

### Screening Population

The analysis focused on adults >18 years years of age. Because the prevalence of CQF is not uniformly distributed in the population (most QF patients resided in the south of the Netherlands; patients can have risk factors for CQF), we considered different subgroups for screening. We used the Netherlands population data from 2017 (*15*). First, we stratified the population on the basis of residence area between high, middle, and low QF incidence areas. For this stratification, we used spatial data on QF notifications and farms with QF outbreaks during the epidemic period (2007–2010). Next, we further divided these subgroups on the basis of a risk factor for CQF between persons with a cardiovascular risk factor, an immunocompromised status, or an unknown risk status. The last group was labeled as unknown because the prevalences of heart valve disorders and aortic aneurysms are underreported. Because these cardiovascular prevalences increase with age, the unknown subgroup was split between persons <60 years and >60 years of age. Thus, we considered 12 (3 × 4) subgroups (Table 1). We obtained prevalences of diagnosed and undiagnosed risk factors from the literature (*16**–**21*) (Appendix Table 1).

**Table 1:** Subgroup criteria in a study of the cost-effectiveness of screening for CQF, the Netherlands, 2017*

Category	Condition
Area of residence	
High incidence	>50 acute QF notifications/100,000 inhabitants *and* >2 acute QF notifications OR presence of a farm with QF abortion waves† within a 5-km range during the epidemic period.
Middle incidence	10–49 acute QF notifications/100,000 inhabitants *and* >2 acute QF notifications OR presence of a farm that tested positive in the mandatory bulk tank milk monitoring initiated during the QF epidemic.
Low incidence	<10 acute QF notifications/100,000 inhabitants OR <2 notifications during the epidemic period.
Preexisting risk factor	
Diagnosed cardiovascular risk factor	Heart valve disorder (all types of defects), heart valve prosthesis, aortic aneurysm, prosthesis/stent, history of endocarditis and congenital heart anomalies.
Immunocompromised patients	HIV infection, asplenia, spleen disorder, malignancy or bone marrow transplantation, and patients using immunosuppressant drugs. As proxy for patients using immunosuppressant drugs, prevalence data were used of rheumatoid arthritis patients and patients with inflammatory bowel disease, assuming these patients frequently use immunosuppressant medication.
Unknown, >60 y	Age >60 y AND no or undiagnosed cardiovascular risk factor, e.g., heart valve disorder, aortic aneurysm.
Unknown 18–59 y	Age 18–59 y AND no or undiagnosed cardiovascular risk factor, e.g., heart valve disorder, aortic aneurysm.

*The epidemic period was 2007–2010. QF, Q fever. †Abortion of >5% of pregnant goats in a farm over a 4-week period.

### Model

We used a decision-tree model that consisted of 2 parts: a screening part and a clinical part (Appendix Figure 1). CQF is usually characterized by persistent high IgG against *C. burnetii* phase I, often in the presence of high IgG against phase II (*2*,*3*). In the current clinical setting in the Netherlands, patients suspected of having CQF are tested with immunofluorescence assay (IFA) for IgG against phase I. However, IFA is a nonautomated and subjective test, and its use might not be feasible for a large-scale screening program (*22*). Therefore, we proposed an initial screening round with the ELISA for IgG against phase II, and positive samples were tested with IFA for IgG against phase I. In the sensitivity analysis, we explored a scenario with direct testing with IFA for IgG against phase I.

In the clinical part, patients were first classified among proven, probable, or possible CQF, according to the guideline of the Dutch Q Fever Consensus Group (*23*). This classification ranks the probability of having CQF based on PCR, serology, clinical parameters, imaging techniques, and pathologic findings (Appendix Table 2). Next, patients were divided by focus of infection and whether CQF led to an early complication (before diagnosis or within 12 weeks after diagnosis). Complications considered were heart failure, symptomatic aneurysm, arterial embolic complication, and other complications. After diagnosis, antimicrobial treatment can be initiated, possibly combined with a surgical procedure. Then, patients may have a late complication (>12 weeks after diagnosis) and can die of CQF.

### CQF Prevalence

The prevalence of CQF 7 years after the QF epidemic is uncertain because the average duration between infection and development of CQF is unknown. Therefore, we considered 2 scenarios, a low CQF prevalence scenario and a high CQF prevalence scenario. For both scenarios, we estimated the prevalence of CQF in 3 consecutive steps: 1) define the risk for *C. burnetii* infection per QF incidence area, 2) multiply by the risk for CQF given infection per risk group, and 3) adjust the CQF prevalence from directly after the epidemic to the year of screening 7 years later. This final step accounts for a decrease of CQF prevalence over time, for instance, because of death or earlier diagnosis.

We selected parameter values for the low and high CQF prevalence scenarios (Table 2). In the low CQF prevalence scenario, we assumed that only patients with a *C. burnetii* infection during the epidemic period were at risk for CQF. We divided them among high, middle, and low QF incidence areas using small geographic areas (4-digit postal code) and used incidence rates of QF notifications during the epidemic period for each incidence area. To adjust for underreporting, we multiplied the incidence rates by 12.6 (*7*). In the high CQF prevalence scenario, we assumed that all patients who seroconverted after the epidemic can develop CQF. For this scenario, we used larger geographic areas (3-digit postal code areas) and *C. burnetii* seroprevalences for each incidence area from the literature (*24*,*25*). In the second step, we estimated the risk for CQF using targeted screening studies for CQF conducted during or immediately after the epidemic (Appendix Table 4) (*9**–**11*,*26*,*27*). In the third step, we based the adjustment of the CQF prevalence from directly after the epidemic to the year of screening for the low CQF prevalence scenario on the reduction of CQF patients in the national CQF database over time (*28*). For the high prevalence scenario, we estimated this adjustment factor on the risk for CQF among patients with a heart valve disorder in studies conducted immediately after the outbreak (*9*,*10*) and a study conducted in 2016–2017 (*29*) (Appendix).

**Table 2:** Prevalence scenarios explored in a study of the cost-effectiveness of screening for CQF, the Netherlands, 2017*

Parameter	Low CQF prevalence scenario	High CQF prevalence scenario
Risk for *Coxiella burnetii* infection	Based on incidence rates of new infections during the epidemic period, adjusted for underreporting	Based on overall seroprevalences from the literature (*24*,*25*)
High incidence area, %	2.15	10.7
Middle incidence area, %	0.15	2.30
Low incidence area, %	0.027	1.00
Risk for CQF after *C. burnetii* infection	Equal for low and high CQF prevalence scenarios. Risk for CQF after infection is 7% for patients with heart valve disorders/prostheses, 29.3% for patients with vascular disorders/prostheses, and 6.9% for immunocompromised patients (probable or proven CQF). Risk for possible CQF in patients without risk factor is 0.2%.	
Adjustment factor to account for reduction of CQF prevalence from directly after epidemic (2010–2012) to year of screening (2017)	0.25	0.52

*The epidemic period was 2007–2010. CQF, chronic Q fever.

### Detection Rate of Screening and Regular Care

We assumed a participation rate in the screening program of 50%, which is the lower bound of previous targeted screening programs for CQF in the Netherlands (*10*,*27*,*30*). The prevalence of CQF was assumed to be equal between participating and nonparticipating persons; hence, the participation rate affects only the number of CQF patients detected but not the cost-effectiveness of screening. We obtained sensitivity and specificity of ELISA from the literature; these values accounted for decreasing sensitivity over time after infection (*31*) (Appendix Table 5). CQF patients with high IgG against phase I were assumed to also have high IgG against phase II (C.C.H. Wielders, unpub. data [*32*]), which implies that all CQF patients test positive with ELISA. In the second screening round using IFA, patients with an IgG >1:512 against phase I were clinically evaluated. The detection rate of CQF in regular care is unknown; we used a detection rate of 80% for proven CQF, 50% for probable CQF, and 10% for possible CQF.

### Outcome Probabilities

We estimated outcome probabilities using data from the national CQF database (Appendix Table 6). This database contains information about 439 CQF patients in the Netherlands, of whom 249 had proven, 74 had probable, and 116 had possible CQF (*6*). To estimate the effectiveness of screening, we stratified outcome data between CQF patients detected by regular healthcare (358 patients) and CQF patients detected by screening (78 patients). Proven CQF patients detected through screening had a 4.0 (95% CI 3.3–4.7) times lower risk for an early complication, 2.8 (95% CI 2.2–3.3) times lower risk for surgery, and 1.8 (95% CI 1.1–2.5) times lower risk for CQF-related death compared with proven CQF patients detected through regular care. The risk for a late complication did not differ significantly (risk ratio 0.7 [95% CI 0.1–1.4]) and was assumed to be equal between screening and regular care. For probable CQF patients, outcome probabilities were not significantly lower for screened patients than for patients identified through regular care. To avoid overestimation of the effect of screening, we conservatively assumed no effectiveness of screening for probable CQF patients and explored a scenario in which probable CQF patients benefit from screening in the sensitivity analysis. No clinical events were assumed in possible CQF patients (*6*). For undetected CQF patients, we used a higher risk for a late complication and death than for patients found through regular care.

### QALYs and Costs

We estimated QALYs by multiplying the utility value associated with a certain health status by the years lived in that status. We obtained utility data for CQF-related complications from the literature (*33**–**36*) (Appendix Table 7). We applied a disutility for antimicrobial treatment (*37*,*38*). Average life expectancies of patients with premature CQF-related death were obtained from the national CQF database (*6*) (Appendix Table 8). For patients without premature CQF-related death, we assumed life expectancy to be half the life expectancy of a person at that age from the general population (*39*). We also obtained utility values for the general population from the literature (*40*) (Appendix).

We calculated costs in 2016 Euros (Appendix Table 9). Direct healthcare costs include costs of screening, diagnostic procedures, surgical procedures, antimicrobial drugs, specialist consultations, and lifelong costs of chronic complications. According to the national cost-effectiveness guideline (*41*), indirect healthcare costs (healthcare costs unrelated to CQF in life-years gained) should be taken into account, which we estimated using a prespecified tool (*42*). Because guidelines from other countries do not consider indirect healthcare costs, we show results without including indirect healthcare costs in the sensitivity analysis. Direct nonhealthcare costs include travel costs, and indirect nonhealthcare costs include productivity losses resulting from work absence (Appendix).

### Cost-effectiveness and Sensitivity Analysis

We calculated the incremental cost-effectiveness ratio (ICER) of screening versus no screening by dividing the difference in costs by the difference in QALYs. We conducted a multivariate probabilistic sensitivity analysis using 10,000 simulations in which we varied a set of parameters at the same time within their uncertainty distributions. We conducted univariate sensitivity analyses, in which we varied several parameters one by one.

## Results

### CQF Prevalence

**Figure 2:**
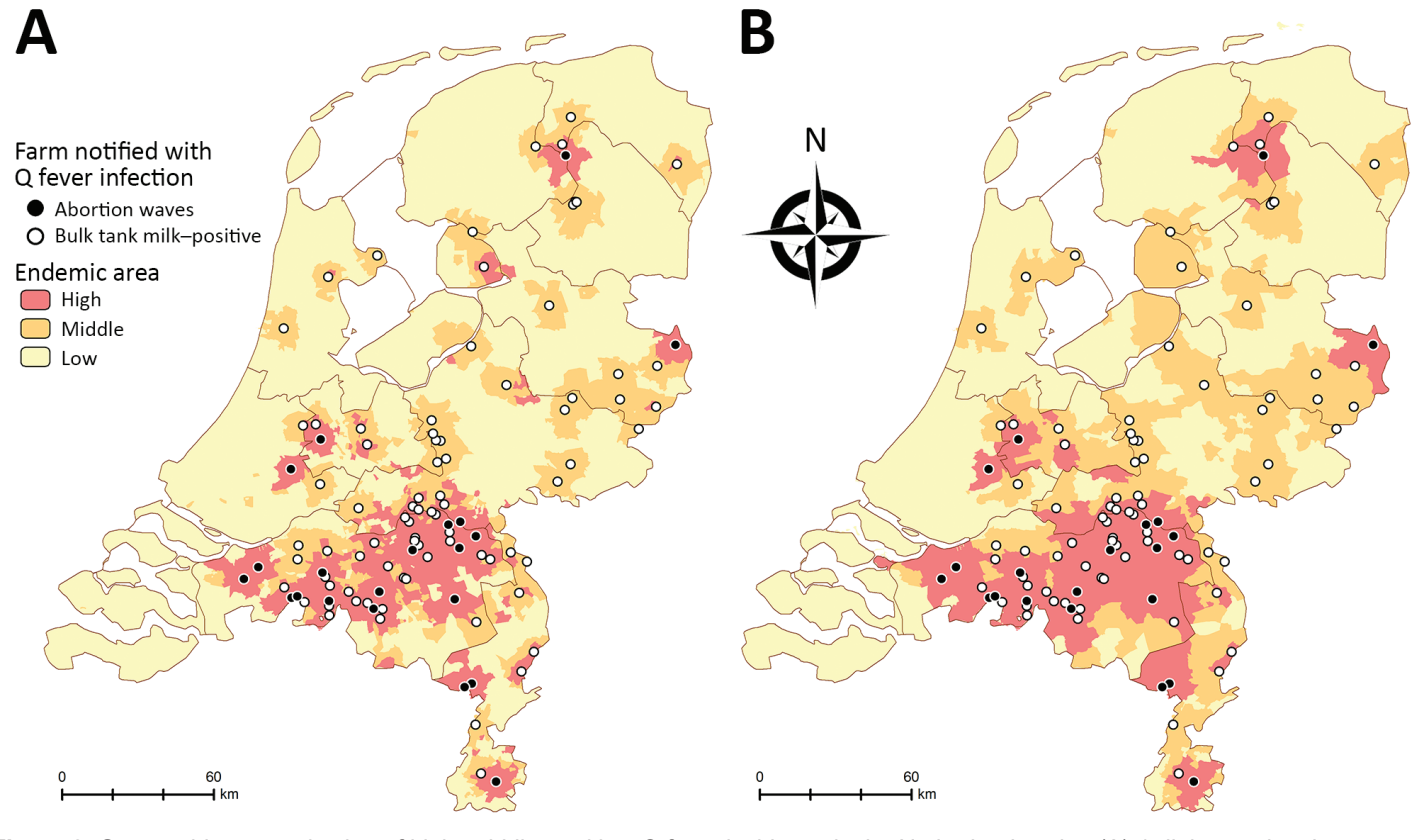
Geographic categorization of high, middle, and low Q fever incidence in the Netherlands using (A) 4-digit postal code areas and (B) 3-digit postal code areas. Incidence level was based on acute Q fever notifications and the proximity of farms with Q fever during the epidemic period (2007–2010).

Depending on the size of the areas, 12% of the population (3-digit postal codes) or 16% of the population (4-digit postal codes) live in high QF incidence areas (Figure 2; Appendix Table 10). For the low CQF prevalence scenario, we estimated the number of *C. burnetii* infections at 42,143, resulting in 414 CQF patients directly after the epidemic and 102 CQF patients in the year of screening. For the high CQF prevalence scenario, the number of *C. burnetii*–infected persons was estimated to be 391,188, resulting in 3,842 CQF patients directly after the epidemic and 1,844 CQF patients in 2017. We also stratified the population by risk factor (Appendix Table 11). The prevalence of CQF varied substantially among risk groups and by residence area (Table 2); the highest prevalence occurred in cardiovascular risk patients living in high incidence areas (Appendix Table 12).

### Clinical Impact

We determined the number of CQF patients and prevented clinical events for each subgroup (Table 3; Appendix Tables 13, 14). Most CQF-related events are prevented by screening of cardiovascular risk groups living in high incidence areas. At an assumed participation rate of 50%, 8 complications, 4 surgeries, and 2 premature deaths are prevented for the low CQF prevalence scenario and 105 complications, 54 surgeries, and 26 premature deaths for the high CQF prevalence scenario. Screening of immunocompromised patients or all adults >60 years of age living in high-risk incidence areas, or screening of cardiovascular risk groups in middle-incidence areas, also could prevent a substantial number of clinical events.

**Table 3:** Outcomes of screening for chronic Q fever when a participation rate of 50% was assumed, the Netherlands, 2017*

Target population	CQF prevalence scenario	CQF prevalence	Persons screened	CQF patients detected	Proven CQF patients detected	Complications prevented	Surgeries prevented	Deaths prevented	QALYs gained	Total cost difference, €, millions	ICER, €/QALY gained
High incidence area											
CVRF patients	Low	644	27,911	18.0	12.4	8.4	4.3	2.1	17.1	0.54	31,737
	High	6,245	36,098	225.4	155.4	104.7	53.9	25.8	214.9	−0.07	Cost-saving
Immunocompromised patients	Low	364	26,898	9.8	6.7	4.5	2.3	1.1	9.3	0.62	66,145
	High	3,525	34,789	122.6	84.5	56.9	29.3	14.0	116.9	0.27	2,312
Age >60 y, unknown risk factor	Low	41.6	219,247	9.1	4.8	3.2	1.6	0.8	6.6	4.46	679,136
	High	305	283,564	86.4	59.6	40.1	20.7	9.9	82.4	5.70	69,208
Age 18–59 y, unknown risk factor	Low	11.0	551,381	6.1	0.2	0.1	0.1	0	0.2	16.23	76,308,665
	High	3.9	713,133	2.8	1.9	1.3	0.7	0.3	2.7	21.41	8,029,064
Middle incidence area											
CVRF patients	Low	45.5	44,586	2.0	1.4	0.9	0.5	0.2	1.9	0.96	495,918
	High	1,342	61,503	82.6	56.9	38.3	19.7	9.4	78.7	1.02	12,929
Immunocompromised patients	Low	25.7	42,969	1.1	0.8	0.5	0.3	0.1	1.1	1.04	990,755
	High	758	59,273	44.9	30.9	20.9	10.7	5.1	42.8	1.23	28,755
Age >60 y, unknown risk factor	Low	2.9	350,237	1.0	0.5	0.4	0.2	0.1	0.7	7.12	9,610,222
	High	65.5	483,129	31.7	21.8	14.7	7.6	3.6	30.2	9.80	324,632
Age 18–59 y, unknown risk factor	Low	0.78	880,807	0.7	0	0	0	0	0	25.83	1,077,459,984
	High	0.84	1,215,017	1.0	0.7	0.5	0.2	0.1	1.0	35.80	36,661,479
Low incidence area											
CVRF patients	Low	8.2	158,759	1.3	0.9	0.6	0.3	0.1	1.2	3.43	2,757,608
	High	584	133,654	78.0	53.8	36.2	18.7	8.9	74.4	2.60	34,912
Immunocompromised patients	Low	4.64	153,001	0.7	0.5	0.3	0.2	0.1	0.7	3.72	5,495,846
	High	329	128,807	42.4	29.2	19.7	10.1	4.9	40.5	2.93	72,544
Age >60 y, unknown risk factor	Low	0.53	1,247,109	0.7	0.3	0.2	0.1	0.1	0.5	25.35	53,126,291
	High	28.5	1,049,899	29.9	20.6	13.9	7.2	3.4	28.5	21.32	747,603
Age 18–59 y, unknown risk factor	Low	0.14	3,136,344	0.4	0	0	0	0	0	91.94	5,955,497,518
	High	0.37	2,640,382	1.0	0.7	0.4	0.2	0.1	0.9	77.57	84,075,394

*Results are stratified by target population and prevalence. CVRF, cardiovascular risk factor; QALY, quality-adjusted life year. †Per million population.

### Cost-effectiveness

We determined the incremental costs, incremental QALYs, and ICERs for each subgroup (Table 3; Appendix Tables 15–17). The ICER of screening of cardiovascular risk groups living in high QF incidence areas was €31,737 per QALY for the low CQF prevalence scenario and cost-saving for the high CQF prevalence scenario. The next most cost-effective strategy would be screening of immunocompromised patients living in high incidence areas; ICERs were €66,145 per QALY for the low CQF prevalence scenario and €2,312 per QALY for the high CQF prevalence scenario. The ICER of screening for cardiovascular risk groups would increase substantially outside the high QF incidence area. For the high CQF prevalence scenario, the ICER increased from cost-saving to €12,929 per QALY in middle QF incidence areas and to €34,912 per QALY in low QF incidence areas. The ICER of screening for adults >60 years of age with an unknown risk factor living in high QF incidence areas was €679,136 per QALY in the low CQF prevalence scenario and €69,208 per QALY in the high CQF prevalence scenario. Screening of adults 18–59 years of age with an unknown risk factor was at least €8 million per QALY.

### Sensitivity Analysis

**Figure 3:**
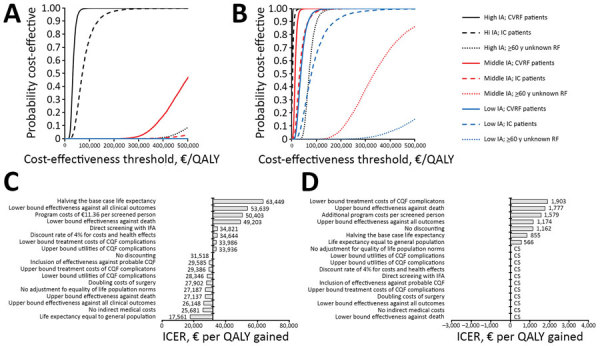
Sensitivity analysis of a screening program for CQF 7 years after the 2007–2010 epidemic, the Netherlands. A, B) Results of the multivariate probabilistic sensitivity analysis of screening in various target groups for a low CQF prevalence scenario (A) and a high CQF prevalence scenario (B). C, D) Results of a univariate sensitivity analysis of screening for chronic Q fever in patients with CVRFs living in high incidence areas for a low CQF prevalence scenario (C) and a high CQF prevalence scenario (D). CQF, chronic Q fever; CVRF, cardiovascular risk factor; IA, incidence area; IC, immunocompromised; ICER, incremental cost-effectiveness ratio; IFA, immunofluorescence assay; QALY, quality-adjusted life year; RF, risk factor.

We conducted a multivariate probabilistic sensitivity analysis (Figure 3; Appendix Figure 2). In the low CQF prevalence scenario, screening of cardiovascular risk patients living in high incidence areas had a 3.1% chance of an ICER <€20,000 per QALY and 92.5% chance of an ICER <€50,000 per QALY (Figure 3, panel A). In the high CQF prevalence scenario, screening had a 54.4% chance of being cost-saving and 100% chance of an ICER <€20,000 per QALY (Figure 3, panel B) for this subgroup.

The ICER was most sensitive to the lifetime costs of complications, the life expectancy of CQF patients, and the effectiveness of the screening program. For the low CQF prevalence scenario, the ICER varied from €17,561 to €63,449 per QALY (Figure 3, panel C). Adding the effectiveness of screening for probable CQF patients changed the ICER from €31,737 to €29,585 per QALY. Exclusion of indirect healthcare costs reduced the ICER to €25,681 per QALY (ICERs without the inclusion of indirect healthcare costs of other subgroups are shown in Appendix Table 18). Adding additional program costs of €11.36 per participant increased the ICER to €53,639 per QALY. For the high CQF prevalence scenario, the ICER remained cost-saving in most scenarios explored, and the highest ICER found was €1,903 per QALY (Figure 3, panel D).

## Discussion

We assessed the cost-effectiveness of a 1-time screening program for CQF in the Netherlands 7 years after a large QF epidemic. Cost-effectiveness varied substantially among areas and risk groups, and the results are highly sensitive to the prevalence of CQF. In a high CQF prevalence scenario, screening of cardiovascular risk patients living in high QF incidence areas during the epidemic was estimated cost-saving, whereas in a low CQF prevalence scenario the ICER was €31,737 per QALY for this subgroup. We found substantially higher ICERs for screening in areas with lower QF incidence during the epidemic or for screening of adults with an unknown risk factor for CQF.

A limitation is that the true prevalence of CQF 7 years after the epidemic is unknown. This prevalence can be affected by many factors, such as death from CQF or other causes, earlier diagnosis in regular care, and the background QF incidence after the epidemic. To account for uncertainty in CQF prevalence, we conducted a low and high CQF prevalence analysis. The estimated 42,000 new *C. burnetii* infections and 411 CQF patients during or after the epidemic low CQF prevalence scenario estimated correspond with previous estimates from the literature (*7*) or CQF patients included in the national database until May 2016 (*6*). However, these numbers are thought to be the absolute minimum. Only 23% of the proven CQF patients had a diagnosed acute QF episode (*6*), and a postmortem study among patients with a history of heart valve surgery in the epidemic area indicates that CQF possibly contributed to the death in 15% of the patients (*9*). The high CQF prevalence scenario could be the upper range because it does not account for preexisting immunity from before the epidemic. It is therefore likely that the true prevalence falls within the reported ranges.

Recent seroprevalence studies performed outside high QF incidence areas are lacking. Underreporting of QF could be higher in these areas because medical doctors are less familiar with QF symptoms (*7*). Furthermore, the geographic division between high, middle, and low QF incidence areas is arbitrary. Persons could be infected while traveling, and the extent to which extent farms with positive bulk milk samples contribute to disease spread is uncertain because 1 infected goat could yield a positive result.

The effectiveness of screening on the prevention of CQF-related complications and premature death is not well documented. We estimated the effectiveness by comparing outcome data between patients detected by screening and by regular care. We did this comparison separately for different CQF categories (proven, probable, or possible), but the effectiveness of screening can still be biased by uncontrolled confounders, such as age and presence of underlying conditions. The effectiveness of antimicrobial treatment for CQF has never been assessed in a randomized clinical trial. Surgery is known to have a positive effect on survival of CQF patients with vascular infection (*3*).

Our cost-effectiveness analysis is based on data from several sources in the Netherlands, such as spatial data on notifications of acute QF, seroprevalence data of *C. burnetii* infections, risk factor–specific probabilities of CQF given infection, and clinical data from a large number of CQF patients. However, combining data from different sources could also introduce biases when study populations do not exactly overlap or screening studies are conducted at different time-points.

Results of our study could also be relevant for other countries, where CQF also might be underreported. For instance, the seroprevalence of *C. burnetii* infection in the United States was estimated at 3.1% (*43*), representing millions of infections and potentially thousands of CQF cases, but no high numbers of CQF have been reported. An explanation may be that *C. burnetii* infections in the United States originate from cattle. The *C. burnetii* strains circulating in cattle differ from and are considered less pathogenic than the strains in small ruminants (*3*). In France, however, *C. burnetii* causes 5% of all endocarditis (*44*), and in Israel, *C. burnetii* infection was found in 9% of patients undergoing valve surgical procedure caused by endocarditis (*45*).

Cost-effectiveness is not the only criterion in deciding whether a screening program is justified (*12*). Screening for CQF is based on an antibody profile suggesting a chronic infection but cannot always be linked to a focus of infection (probable or possible CQF patients). Therefore, physicians must make difficult decisions about whether long-term antimicrobial treatment should be initiated when the outcome is uncertain and adverse events frequently occur. Raoult (*46*) has recently proposed alternative definition criteria for CQF from the consensus guideline in the Netherlands; these criteria could exclude most probable and possible CQF patients from follow-up but also may be less sensitive in the diagnosis of proven CQF (*47*).

When screening for CQF would be limited to subgroups for which screening is most cost-effective, a substantial proportion of CQF patients will remain undetected. Serologic follow-up for patients with acute QF is therefore recommended, even in absence of a risk factor for CQF (*32*). However, compliance with this recommendation was suboptimal during the epidemic (*48*), and many patients experience an acute infection asymptomatically or do not have the infection diagnosed. Alongside a standalone screening program, case finding could be implemented in regular care, in which the physician decides whether a patient should be screened according to a risk profile. Also, a combination of case-finding and screening programs among high-risk groups could be initiated; this approach has also been suggested for hepatitis B and hepatitis C (*49*).

Appendix Additional methods and results for study of the cost-effectiveness of screening for chronic Q fever, the Netherlands, 2017.
