# Supplementary material for: Cost-effectiveness of Screening Program for Chronic Q Fever, the Netherlands
Source: Emerg Infect Dis. 2020 Feb;26(2):238–46. doi: 10.3201/eid2602.181772 (PMC6986831; doi:10.3201/eid2602.181772)
Supplement: Appendix — Additional methods and results for study of the cost-effectiveness of screening for chronic Q fever, the Netherlands, 2017. [file 18-1772-Techapp-s1.pdf]

Article DOI: https://doi.org/10.3201/eid2602.181772

Cost-Effectiveness of Screening Program for Chronic Q Fever, the Netherlands

Appendix

Supplemental Methods

Prevalence of Risk Factors for Chronic Q Fever

Prevalence rates of risk factors are shown in Appendix Table 1. Prevalence rates of cardiovascular risk factors by age group were based on data from a general practice research database in the Netherlands (*1*). We used prevalence data of patients with heart valve defect, aortic aneurysm/prosthesis, congenital heart anomaly, and endocarditis. As patients can have >1 risk factor, we used prevalence rates of any of these diagnosed cardiovascular risk factors and assigned these patients to the individual cardiovascular risk factors in proportion with the prevalence rates of the risk factor–specific prevalence rates. As the prevalence of aortic aneurysms and heart valve disorders are underreported, we also considered people with undiagnosed cardiovascular risk factors to be at increased risk of chronic Q fever (CQF). Prevalence rates of these undiagnosed cardiovascular risk factors were based on screening studies in the general population, and prevalence rates of diagnosed risk factors were then subtracted from these. For heart valve disorders, we used prevalence rates of clinically relevant heart valve disorders in ≥65-year-olds from the UK (*2*), and for aortic aneurysms, we used prevalence rates of abdominal aortic aneurysms in ≥55-year-olds from the Netherlands (*3*).

The prevalence of patients being immunocompromised due to an underlying disease by age was obtained from a study in the UK and includes patients with HIV infection, asplenia, spleen dysfunction, malignancy (e.g., leukemia), or bone marrow transplant (*4*). As proxy for the prevalence of immunosuppressive drug users, we used prevalence rates by age of rheumatoid arthritis and inflammatory bowel disease (*5,6*). These are the largest patient groups that use immunosuppressive drugs, and we assumed that all these patients use these drugs continuously or have used these drugs at least temporarily. To avoid counting patients twice, we adjusted the

prevalence rates of immunocompromised patients for the probability of having a cardiovascular risk factor. As the risk of developing CQF in patients with cardiovascular risk factors is thought to be higher than in immunocompromised patients (*7)*, we considered patient with both a cardiovascular risk factor and an immunocompromised status in our model as a patient with cardiovascular risk factor.

Appendix Table 1. Prevalence of risk factors for chronic Q fever (per 10,000 persons)

	Age group, y								
Population	18–19	20–29	30–39	40–49	50–59	60–69	70–79	80–89	>90
Diagnosed cardiovascular risk factor	74	66	60	132	171	476	948	1,666	1,845
Heart valve disorders or =prosthesis	14	19	28	87	122	373	793	1,375	1,760
Aortic aneurysm or -prosthesis	5	4	0	11	34	85	222	339	172
Congenital heart anomaly	70	51	34	39	25	31	7	35	0
Endocarditis	0	2	6	6	13	27	21	28	0
Undiagnosed cardiovascular risk factor									
Heart valve disorder*	0	0	0	0	0	57	251	941	1,220
Aortic aneurysm†	0	0	0	0	10	101	120	194	255
Immunocompromised									
Underlying disease‡	90	90	90	90	90	158	230	230	230
Medication use									
Rheumatoid arthritis	21	39	68	115	177	273	353	465	507
Inflammatory bowel disease	14	39	32	35	46	81	119	95	95

*Only clinically relevant heart valve disorder.
†Abdominal aortic aneurysms only.
‡Includes HIV infection, asplenia, spleen dysfunction, malignancy (e.g., leukemia), or bone marrow transplant.

Model Design

Appendix Figure 1 shows the decision tree of the screening par (panel A) and the clinical part (panel B).

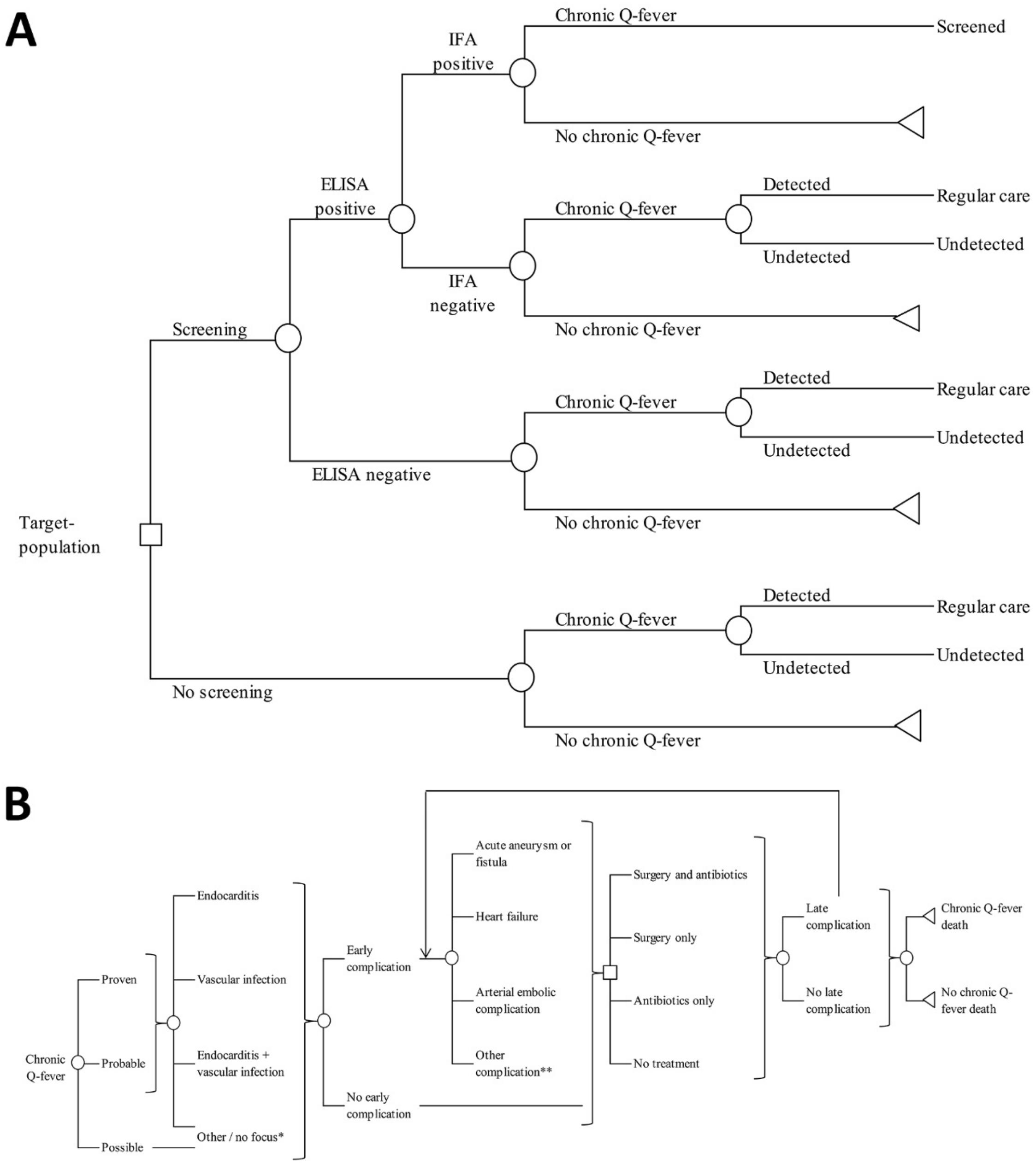

Appendix Figure 1. Decision tree model. A) Decision tree for detection of chronic Q fever in presence or absence of a screening program. A square represents a decision node, a circle represents a chance node, and a triangle represents a terminal node. IFA, Immunofluorescence assay. B) Decision tree for the clinical outcomes of chronic Q fever after screening, regular care, or undetected (outcome of the decision tree of screening). * contains less prevalent presentations, i.e., osteomyelitis, pericarditis, and spondylodiscitis. ** includes non-cardiac abscess, spondylodiscitis and osteomyelitis.

Definition of Chronic Q Fever

Appendix Table 2 shows the definition of chronic Q fever according to the Dutch Q fever consensus group (*8*).

Appendix Table 2. Diagnostic criteria for CQF as defined by the Dutch Q Fever Consensus Group*

Category	Criteria
Proven CQF	1) Positive *Coxiella burnetii* PCR in blood or tissue in absence of an acute Q fever infection OR<br>2) IFA >1:1,024 for *C. burnetii* phase I IgG, AND >1 of the following criteria:<br>- Definite endocarditis according to the modified Duke criteria (*9*) OR<br>- Proven large vessel or prosthetic infection, confirmed by imaging studies (e.g., PET-CT).
Probable CQF	IFA >1:1,024 for *C. burnetii* phase I IgG AND >1 of the following criteria:<br>- Valvulopathy not meeting the major criteria of the modified Duke criteria (*9*).<br>- Known aneurysm or vascular or cardiac valve prosthesis without signs of infection (by means of TEE/TTE, PET-CT, other imaging studies).<br>- Suspected osteomyelitis, pericarditis or hepatitis as manifestation of CQF.<br>- Pregnancy.<br>- Symptoms and signs of chronic infection, such as fever, weight loss and night sweats, hepato-splenomegaly, persistent raised ESR and CRP.<br>- Granulomatous tissue inflammation proven by histologic examination.<br>- Immunocompromised state.
Possible CQF	IFA >1:1,024 for *C. burnetii* phase I IgG without meeting the criteria for proven or probable CQF

*CQF, chronic Q fever; CRP, C-reactive protein; ESR, erythrocyte sedimentation rate; IFA, immunofluorescence assay; PET-CT, positron emission tomography–computed tomography; TEE, transesophageal echocardiography; TTE, transthoracal echocardiography.

Prevalence of Chronic Q Fever

We estimated the prevalence of CQF in 3 steps:

1) Estimating the number of patients with a *Coxiella burnetii* infection. This was done separately for high, middle, and low QF incidence areas during the epidemic.

2) Estimating the number of patients that develop CQF after *C. burnetii* infection. This was separately done for risk groups (heart valve disorder, aortic aneurysm, compromised immune system, or none of the aforementioned risk factors).

3) Estimating the number of CQF patients that are still alive and undetected in the year screening 7 years after the epidemic.

Given the uncertainty around the prevalence of CQF 7 years after the epidemic, we analyzed 2 scenarios: 1) a low prevalence scenario and 2) a high prevalence scenario.

Estimating the Number of Patients with a *C. burnetii* Infection

The low prevalence scenario assumes that only patients infected with *C. burnetii* during the epidemic (period 2007–2010) are able to develop CQF; hence, individuals that were seroconverted before the epidemic only had an immune boost but no risk of developing CQF.

These boosted individuals are treated as seronegative in the model. The risk of a *C. burnetii* infection during the epidemic is based on Dutch incidence rates of QF notifications for areas that were qualified as high, middle, and low incidence area. The distribution of the population between high, middle, and low incidence areas was estimated using the incidence of QF notifications and the proximity of a farm with QF abortion waves or the proximity of a farm that tested positive in the mandatory bulk tank milk monitoring within a range of 5 km during the epidemic (Table 1 in the main article for more details). To account for underreporting because of asymptomatic infections or symptomatic infections that were not medically attended or diagnosed, we multiplied these notification rates by 12.6. This multiplication factor was based on a study from the Netherlands that compared QF notification rates with seroconversion rates in blood donors from whom serial samples were available (*10*). The adjusted risk of *C. burnetii* infection during the epidemic was then estimated at 2.15% in high incidence areas, 0.15% in middle incidence areas, and 0.027% in low incidence areas.

In the high prevalence scenario, the risk of *C. burnetii* infection was based on Dutch seroprevalence studies. This scenario assumes that all patients tested seropositive after the epidemic are able to develop CQF, independent whether they were already infected before the epidemic and immune during the epidemic or not. The seroprevalence in high incidence areas was estimated at 10.7%. This estimate was based on a large seroprevalence study in areas with high QF incidence during the epidemic in 2014–2015 finding a seroprevalence of 6.0%. However, the used ELISA test for IgG phase II is known to decrease over time and the seroprevalence study was conducted 5 years after the epidemic in 2007–2010. Follow-up data over 4 years showed a decreasing trend of ELISA sensitivity after *C. burnetii* infection over time (C.C.H. Wielders, unpub. data from [*10*]) and, after extrapolation of this decreasing to 5 years after *C. burnetii* infection using a lognormal curve, we found that 55.9% of the patients test would still test positive after 5 years. We adjusted the seroprevalence to 10.7% using longitudinal data on sensitivity of. In absence of serologic studies in middle and low incidence areas, we used data from a study that measured the seroprevalence of *C. burnetii* using IFA for IgG phase II in an area that covered high, middle, and low incidence areas in 2008 (before the epidemic in this part of the country) and in 2010 (the final year of the QF epidemic). The seroprevalence of 3.2% after the epidemic was used for middle incidence areas and the

seroprevalence of 1.0% before the epidemic was used for low incidence areas. More details of the studies are listed in Appendix Table 3.

Not relevant for the cost-effectiveness within a specific incidence area, but relevant for the absolute number of cases, is the size of the areas that are divided between high, middle, and low incidence areas. For the low prevalence scenario, we based this division based on 4-digit postal code areas and for the high prevalence scenario we used 3-digit postal codes (larger areas). Use of 4-digit postal code areas result in a lower number of infections, as the areas that are assigned to high or moderate incidence areas due to the proximity of an infected farm are smaller.

Estimating the Number of Patients that Develop CQF after *C. burnetii* Infection

The second step of estimating the risk of developing CQF after *C. burnetii* infection was assumed to be equal for the 2 prevalence scenarios. The risk of CQF given *C. burnetii* infection in risk groups was based on targeted screening studies for CQF from the Netherlands that were conducted during or directly after the epidemic (Appendix Table 4). Most of these studies defined CQF as an IgG titer of 1:512 or 1,024 against *C. burnetii* phase I or a positive PCR not related to acute QF. The risk of CQF differs by pre-existing risk factor, estimated at 8.7% for patients with heart valve disorders/prostheses (*11,12*), 29.3% for patients with vascular disorders/prostheses (*11,13*), and 6.9% for immunocompromised patients (*14*). In accordance with the Dutch consensus guideline, detected CQF patients in these studies are by definition proven or probable CQF patients because they have a risk factor (*15*). We applied the same risk of CQF for diagnosed and undiagnosed cardiovascular risk factors. For people without a risk factor, we estimated that 0.2% had possible CQF based on a Dutch screening study in the general population (*16*).

Appendix Table 3. Prevalence of *Coxiella burnetii* infection by CQF prevalence scenario and incidence area*

Area	Deterministic	SD†	Distribution†	Source
Low CQF prevalence scenario				
High incidence area	0.0215	95% CI 0.0208–0.0223	Lognormal	Based on the incidence of QF notifications in areas with low QF incidence (see main article Table 1 for criteria) during the period 2007–2010, adjusted for underreporting by multiplying with 12.6 (*10*).
Middle incidence area	0.00152	95% CI 0.00137–0.00168	Lognormal	Based on the incidence of QF notifications in areas with middle QF incidence (see main article Table 1 for criteria) during the period 2007–2010, adjusted for underreporting by multiplying with 12.6 (*10*).
Low incidence area	0.000275	95% CI 0.000243–0.000311	Lognormal	Based on the incidence of QF notifications in areas with low QF incidence (see main article Table 1 for criteria) during the period 2007–2010, adjusted for underreporting by multiplying with 12.6 (*10*).
High CQF prevalence scenario				
High incidence area	0.107	95% CI 0.088–0.131	Lognormal	Pijnacker, 2017 (*17*). The seroprevalence of QF was adjusted from 6.0% to 10.7% to account for a decreasing sensitivity of ELISA over time (unpub. data from [*13*]).
Middle incidence area	0.0230	95% CI 0.0140–0.0380	Lognormal	Brandwacht, 2010 (*18*). Based on seroprevalence data of 2010 in areas of the Netherlands that covered high, middle, and low incidence areas.
Low incidence area	0.0100	95% CI 0.0050–0.0190	Lognormal	Brandwacht, 2010 (*18*). Based on seroprevalence data of 2008 from before the area was affected during the epidemic.

CQF: Chronic Q fever; QF, Q fever.
†Used for the multivariate probabilistic sensitivity analysis.

Appendix Table 4. Dutch screening studies on the risk of chronic Q fever among individuals tested seropositive for *Coxiella burnetii**

Risk condition	Study	Population	Incidence area	Study period	Test and cutoff value	CQF given seropositive for *C. burnetii* infection	% CQF	SD†	Distribution†	Additional information
Screening studies conducted directly after the QF epidemic of 2007–2010										
Aortic aneurysm/prosthesis	Hagenaars, 2014 (*13*)	Patients with abdominal aortic- or ileac aortic aneurysm, or reconstruction	High	2009–2012	IFA IgG phase I ≥1:512	40/130	30.8			
	Wegdam-Blans, 2013 (*12*)	Patients with abdominal aortic aneurysm or vascular prosthesis	High	2010–2011	IFA IgG phase I ≥1:1.024 or positive PCR	7/30	23.1			
	Total					47/160	29.3	0.02	Beta	All proven or probable CQF[a]
Heart valve disorder/prosthesis	Wegdam-Blans, 2013 (*12*)	Patients with heart valve prosthesis	High	2010–2011	IFA IgG phase I ≥1:1.024 or positive PCR	3/22	13.8			
	Kampschreur, 2012 (*11*)	Patients with history of heart valve surgery	High	2010–2011	IFA IgG phase I ≥1:512	9/116	7.8			
	Total					12/138	8.7	0.04	Beta	All proven or probable CQF[a]
Immunocompromised patients	Schoffelen, 2014 (*14*)	Patients with rheumatoid arthritis	High	2011–2012	Not reported	7/102	6.9	0.03	Beta	All proven or probable CQF[a]
Non-risk patients	Morroy, 2015 (*16*)	All adults	High	2014	IFA IgG phase I ≥1:512	1/491[b]	0.2	0.001	Beta	All possible CQF
Screening studies conducted close to the year of the screening in 2017										
Heart valve disorder/prosthesis	De Lange, 2019 (*19*)	Patients with heart valve disorder	High	2016–2017	IFA IgG phase I ≥1:512	6/133	4.5			All proven or probable CQF[a]

*IFA, Immunofluorescence assay; QF, Q fever.
†According to the Dutch consensus guideline patients with risk factors and titer IgG phase I ≥1:512 automatically qualify for probable or proven CQF (*8*). [b]: Patients with a cardiovaular risk factor or immunocompromised status were excluded.

Estimating the Prevalence of CQF Patients in the Year of Screening

The targeted screening studies referred to in the second step were conducted during or directly after the epidemic (2010–2012), while the screening program was assumed to take place in 2017. As the prevalence of CQF is expected to decline over time due to CQF-related mortality or mortality from another cause and due to detection via regular care, we adjusted the prevalence downwards. This adjustment factor was different in the low and high CQF prevalence scenario. In the low prevalence scenario, we based this adjustment factor on the numbers of CQF patients in the Dutch national CQF database over time. This database includes all diagnosed CQF patients in the Netherlands and shows a high number of proven CQF patients reported in 2010–2011, which drops substantially in the year 2012 and remains relatively stable after 2012 (*20*). The adjustment factor was the division of the average annual number of proven CQF cases in the period 2012–2017 by the average annual number of CQF cases in the period 2010–2011, resulting in an adjustment factor of 0.25. For the high prevalence scenario, we compared the risk of proven or probable CQF given *C. burnetii* infection among people with heart valve disorders between screening studies conducted during or directly after the epidemic (*17,18*), and a recent screening study conducted in 2016–2017 (*19*). This resulted in an adjustment factor of 0.52 (4.5%/8.7%; see Appendix Table 4).

Sensitivity and Specificity of Testing

Sensitivity of ELISA for IgG phase II and IFA for IgG phase II and phase I are shown in Appendix Table 5. Sensitivity of ELISA 7 years after the epidemic was estimated by extrapolating longitudinal data on sensitivity of ELISA over the first 4 years after infection (C.C.H. Wielders, unpub. data from [*21*]). The specificity was based on a study from Germany (*22*). Cutoff for ELISA positivity was according to the manufacturer's instruction, considering borderline samples as positive. We assumed that all CQF patients had high IgG phase II titers (C.C.H. Wielders, unpub. data from [*21*]), hence testing positive for ELISA. In the second screening round using IFA, patients were tested for having an IgG titer of ≥1:512 against phase I are clinical examined. As patients with an IgG titer of ≥1:512 against phase I do not necessarily have CQF according to the Dutch consensus guideline (the guideline uses an IgG titer threshold of ≥1:1,024 against phase I). Targeted screening studies in patients with heart valve disorder showed that 8 of 234 patients had an IgG titer of 512 but no CQF (*19,23*), resulting in a

specificity of IFA of 0.966. Similarly, in individuals with no risk factor 2/512 patients had an IgG titer of 512, resulting in a specificity of 0.996 (*16*).

Appendix Table 5. Sensitivity and specificity of ELISA IgG phase II and IFA IgG phase I*

Diagnostic test	Deterministic	SD†	Distribution†	Source
ELISA IgG phase II				
Historic QF only				
Sensitivity	0.50	95% range: 0.39–0.63	Lognormal	Extrapolation of sensitivity data of first 4 y after infection to 7 y after infection (C.C.H. Wielders, unpub. data from [*10*])
Specificity	0.980	0.014	Beta	Frosinski, 2016 (*18*)
CQF				
Sensitivity	1			
IFA IgG phase I titer 1:512				
Proven / probable CQF				
Sensitivity	1			
Specificity	0.966	0.012	Beta	Estimated from Kampschreur 2013 and De Lange 2019 (*19, 23*)
Possible CQF				
Sensitivity	1			
Specificity	0.996	0.003	Beta	Estimated from Morroy, 2016 (*16*)

*CQF, chronic Q fever; QF, Q fever.
†Used for the multivariate probabilistic sensitivity analysis.

Outcome Probabilities of CQF

The outcome probabilities of CQF are listed in Appendix Table 6. The outcome probabilities are stratified by CQF category (proven and probable) and by outcome of the screening decision tree (detected by screening, detected in regular care, not detected at all). Clinical outcome probabilities are obtained from the Dutch national CQF database. Proven and probable patients were stratified between patients detected via screening and patients detected in regular care. We found that proven CQF patients detected by screening had a significantly reduced risk of an early complication, surgery, and CQF-related mortality as compared to patients detected in regular care, but not a significantly reduced risk of a late complication. For probable CQF patients, we found no significant reduction in any clinical outcome. Therefore, we conservatively assumed that screening had no effectiveness against probable CQF. In the sensitivity analysis, we included a scenario in which screening had effectiveness against an early complication. No complications, surgeries, or mortality was reported for possible CQF patients in the national CQF database.

Appendix Table 6. Outcome probabilities of proven or probable CQF*

Parameter	Deterministic	SD†	Distribution†	Scenario	Reference and comments
Classification of proven/probable CQF					
Proven CQF	0.689	0.054	Beta		CQF database (*20*), distribution based on 74 proven and probable CQF patients found via screening.
Probable CQF	0.311				Calculated as 1-proven CQF
Type of infection					
Proven CQF					
Endocarditis	0.273	0.028	Dirichlet		CQF database (*20*), distribution based on 249 proven CQF patients.
Vascular infection	0.502	0.032	Dirichlet		
Endocarditis & vascular infection	0.161	0.023	Dirichlet		
Other /no infection focus	0.064	0.016	Dirichlet		
Probable CQF					
Endocarditis	0.216	0.048	Dirichlet		CQF database (*20*), distribution based on 74 probable CQF patients.
Vascular infection	0.378	0.056	Dirichlet		
Endocarditis & vascular infection	0.041	0.023	Dirichlet		
Other /no infection focus	0.365	0.056	Dirichlet		
Early complication					
Proven CQF					
Late detected by regular care or not detected	0.548	0.04	Beta		CQF database (*20*). Early complication detected in 108/197 patients detected via regular care. Not detected was assumed equal to late detected, as late detected will usually be diagnosed after a complication occurred.
RR due to early detection by screening	3.99	95% CI 3.30–4-69	Lognormal	Lower and upper bound of 95% CI	CQF database (*20*). Early complication in 7/51 patients detected via screening (RR 4.0 [95% CI 3.3–4.7] as compared to detected via regular care)
Early detected by screening	0.137				Probability late detected divided by RR
Probable CQF					
Late detected by regular care or not detected	0.095	0.034	Beta	0.118	CQF database (*20*). Early complication detected in 8/73 patients. Not detected was assumed equal to late detected, as late detected will usually be diagnosed after a complication occurred.
RR due to early detection by screening	1			2.7	No significant difference between patients detected via screening or regular care. (RR 2.7 [95% CI 0.6–4.8])
Early detected by screening	0.095			0.043	Probability late detected divided by RR
Type of complication					
Proven CQF					
Acute aneurysm / fistula	0.542	0.04	Beta		CQF database (*20*). On the basis of 153 complications. Other complications include spondylodiscitis/osteomyelitis and non-cardiac abscess.
Heart failure	0.327	0.04	Beta		
Arterial embolic complication	0.124	0.03	Beta		
Other complication	0.248	0.04	Beta		
Probable CQF					
Acute aneurysm / fistula	0.364	0.15	Beta		CQF database (*20*). On the basis of 11 complications. Other complications include spondylodiscitis/osteomyelitis and non-cardiac abscess.
Heart failure	0.455	0.15	Beta		
Arterial embolic complication	0.091	0.09	Beta		
Other complication	0.091	0.09	Beta		
Surgery					
Proven CQF					CQF database (*20*). Surgery at 107/197 patients detected via regular care and at 10 of 51 detected via screening (RR 2.8 [95% CI 2.2–3.3]).
Late detected by regular care or not detected	0.543				

Parameter	Deterministic	SD†	Distribution†	Scenario	Reference and comments
RR due to early detection by screening	2.77	95% CI 2.20–3.34	Lognormal	Lower and upper bound of 95% CI	
Early detected by screening	0.196				Probability late detected divided by RR
Probable CQF					
Late detected by regular care or not detected	0.081				CQF database (*20*). Surgery at 6/74 patients
RR due to early detection by screening	1				No significant difference between patients detected via screening or regular care (RR 0.5 [95% CI 0–2.0])
Early detected by screening	0.081				Probability late detected divided by RR
Antibiotic treatment initiated					
Proven CQF	0.912	0.02	Beta		CQF database (*20*), 227/249 patients.
Probable CQF	0.662	0.05	Beta		CQF database (*20*), 49/74 patients.
Possible CQF	0				Assumption based on current standard work-up of possible CQF patients (C.P. Bleeker-Rovers, pers. comm.)
Late complication					
Proven CQF					
Not detected	0.452				Assuming that all undetected patients will have a CQF complication; calculated as (1 – probability of early complication)
Late detected by regular care	0.153	0.02	Beta		CQF database (*20*). Late complication in 38/249 patients
RR due to early detection by screening	1				CQF database (*20*). No significant difference between patients detected via screening or regular care (RR 0.7 [95% CI 0.1–1.4]).
Early detected by screening	0.153				Probability late detected divided by RR
Probable CQF					
Not detected	0.095				Assumed equal to early complication.
Late detected by regular care	0.054	0.03	Beta		CQF database (*20*). Late complication in 38/249 probable CQF patients
RR due to early detection by screening	1				CQF database (*20*). No significant difference between patients detected via screening or regular care (RR 1.4 [95% CI 0–3.6].
Early detected by screening	0.054				Probability late detected divided by RR
CQF-related mortality					
Proven CQF					CQF database (*20*). CQF-related mortality at 55/197 proven CQF patients detected via regular care.
Not detected	0.497				Assumed that the RR between non-detected and regular care was equal to between regular care and non-detected. This approximates a 60% death rate among CQF patients in the 1970s, when effective antibiotic treatment was not available and there was a large diagnostic delay (*24*).
Late detected by regular care	0.279	0.032	Beta		CQF database (*20*). CQF-related mortality at 55/197 proven CQF patients detected via regular care.
RR due to early detection by screening	1.78	95% range 1.11–2.45	Lognormal	Lower and upper bound of 95% CI	CQF database (*20*). CQF-related mortality in 8/51 patients detected via screening (RR 1.78 [95% CI 1.11–2.45] as compared to late detected).
Early detected by screening	0.157				Probability late detected divided by RR
Probable CQF					
Late detected by regular care or not detected	0.041	0.023	Beta		CQF database (*20*). CQF-related mortality in 3/74 probable CQF patients
RR due to early detection by screening	1				No significant difference between patients detected via screening or regular care (RR not given due to small numbers)
Early detected by screening	0.041				Probability late detected divided by RR

*CQF, chronic Q-fever; RR, risk ratio.
†Used for the multivariate probabilistic sensitivity analysis.

Quality-Adjusted Life Years

The number of quality-adjusted life years (QALYs) for CQF patients was calculated by multiplying the utilities (preference based measure of health-related quality of life) for each health state with the time spent in that health state.

Utilities

Utilities of the different health states used in this model are shown in Appendix Table 7. As the average age of CQF patient in the national CQF database is 65 years (*25*), we used population norms of $\geq$50-year-olds for the general population (*26*). In a sensitivity analysis we also explored a scenario in which the utility of the general population is 1. Utility data of CQF patients is lacking. Before a complication occurs, CQF is usually asymptomatic or it presents as influenza-like symptoms. We assumed that for proven or probable CQF, the utility is equal to the utility of a patient with a heart valve prosthesis (*27*). We based the utilities of the different health states on quality of life data of the complications. The utility of an aneurysm or fistula was based on patients in need of a surgery for a symptomatic abdominal aortic aneurysm (*28*). The utility of heart failure was based on patients with New York Heart Association class III or IV hear failure (*29*). The utility of patients with an embolic complication was based on patients with a stroke with mild impairment (*30*). We assumed that long-term antibiotic use leads to a reduction of the utility. According to data from France, long-term antibiotic use to treat CQF led to gastrointestinal adverse events in 7% (*24*) of the patients. The disutility of this adverse event was assumed to be 0.105 (*31*). Possible CQF patients were assumed to have no reduction of the utility.

Appendix Table 7. Utilities of the different health states*

Health state	Input	SD†	Distribution†	Scenario	Source
Utilities					
General population	0.857	0.0086	Beta	1	Versteegh, 2016 (*26*)
Proven or probable CQF (uncomplicated)	0.855	0.0051	Beta		Franklin, 2016 (*27*)
Symptomatic aneurysm or fistula	0.690	0.048	Beta		Timmers, 2013 (*28*)
Heart failure	0.610	0.015	Beta		Calvert, 2005 (*29*)
Arterial embolic complication	0.640	0.063	Beta		Stouthard, 1997 (*30*)
Dead	0				
Utility adaption					
Gastroenteritis due to antibiotic use	–0.007	0.0028	Beta		Million, 2010 (*24)*, WHO, 2004 (*31)*

*CQF, chronic Q fever.
†Used for the multivariate probabilistic sensitivity analysis.

Time Spent in Each Health State

Time spent in each health state is shown in Appendix Table 8. It is assumed that patients with a complication remain in the indicated health state for the rest of their lives. The life

expectancy of proven or probable CQF patients with premature CQF-related death was based on survival data of patients included in the Dutch national CQF database (*25*). The life expectancy of patients not dying prematurely due to CQF was based on the life expectancy of a comparable person at that age from the general population. We obtained the average age at diagnosis of proven and probable CQF patients from the national CQF database, being 69 years and 64 years, respectively (*25*). Using lifetables of the Netherlands, the life expectancies in the general Dutch population at these ages are 16.8 years and 20.8 years (*32*). However, the life expectancy of proven and probable CQF patients is expected to be lower than the life expectancy of an average person at that age due to the presence of a cardiovascular risk condition. Based on the comparison of the life expectancy of patients with heart valve prosthesis at the age of 60 years (*33*) with the life expectancy of patients in the general population at that age from the literature, we halved the life expectancy of proven and probable CQF patients to 8.4 years and 10.4 years, respectively. In the sensitivity analysis we explored life expectancies of the general population or halving the base case life-expectancies to 4.2 years for proven CQF and 5.2 years for probable CQF.

For those receiving antibiotic treatment, the duration of treatment was obtained from the national Dutch CQF database for proven and probable CQF patients (*25*).

Appendix Table 8. Time spent in health state*

Outcome	Input	SD†	Distribution†	Scenario	Source
Life expectancy					
CQF-related mortality					
Proven CQF	0.6				Van Roeden, 2018 (*25*)
Probable CQF	2.6				Van Roeden, 2018 (*25*)
No CQF-related mortality					
Proven CQF	8.4			16.8 and 4.2	Average age of diagnosis Van Roeden, 2018 (*25*), life expectancy from Statistics Netherlands (*32*), adjustment factor for co-morbidity from Van Geldorp, 2009 (*33*)
Probable CQF	10.4			20.8 and 5.2	Average age of diagnosis from Van Roeden, 2018 (*25*), life expectancy from Statistics Netherlands (*32*), adjustment factor for co-morbidity from Van Geldorp, 2009 (*33*)
Duration of antibiotic treatment (weeks)					
Proven CQF	96	7.8	Gamma		Van Roeden, 2018 (*34*)
Probable CQF	83	9.1	Gamma		Van Roeden, 2018 (*34*)

*CQF, chronic Q fever.
†Used for the multivariate probabilistic sensitivity analysis.

Costs

In accordance with the Dutch guideline on health economic evaluation in healthcare, we adopted a societal perspective. Costs considered in our analysis are:

- Direct healthcare costs: blood collection, diagnostic tests, surgeries, antibiotics, specialist visits.

- Indirect healthcare costs: costs unrelated to CQF in gained life years of averted premature CQF-related deaths.

- Direct non-healthcare costs: travel costs.

- Indirect non-healthcare costs: Productivity losses due to work absence.

Appendix Table 9 shows the costs inputs presented in 2016 euros (€). Costs from other years were converted to the 2016 price year using the Dutch consumer price index (*35*). A positive ELISA test will be followed by an IFA test for IgG titer of ≥1:512 against phase I (IFA screen) and a positive IFA screen test will be confirmed with a IFA titration to determine the exact titer. Patients with IgG titer of ≥1:512 against *C. burnetii* phase I will then be clinically evaluated by a medical specialist using different serologic tests and imaging techniques (initial diagnostic procedure) whether the patient has proven, probable, or possible CQF. In the base case analysis, we ignored program costs because the screening of risk groups may also occur during routine visits. In the sensitivity analysis, we explored a scenario in which we assumed that the program costs would be €11.36 per screened person for selecting and inviting patients. We based these program costs on the tariff a GP currently receives for the selection, invitation and administration of influenza vaccination within the national influenza immunization program.

Cost of a surgery is the weighted average of vascular surgeries, heart valve surgeries and other kind of surgeries (according to surgery data from S.E. van Roeden, pers. comm., and cost data from the literature [*36,37*]). Surgeries gathered under "other surgeries" mostly consist of the drainage of a non-cardiac abscess and we used the cost of a pulmonary drainage for this parameter. The cost of antibiotics is based on a treatment with doxycycline and hydroxychloroquine and includes also costs of blood tests to determine the antibiotic levels. The duration of antibiotic treatment is shown in Appendix Table 8. During treatment, patients visit the medical specialist every 3 months for serologic follow up, and CQF patients with a vascular infection have a PET scan every year. Follow-up of proven and probable CQF patients is life-long and consists of medical specialist visits and serologic tests of which the frequency reduces over time. Possible CQF patients are followed until the IgG titer against *C. burnetiid* phase I has been decreased to <1:1.,024. We assumed that the average follow-up of possible CQF patients is

1 year. Concerning CQF-related complications, we assumed that the treatment of acute aneurysm, heart failure, and arterial embolic complication would be lifetime. Treatment costs are obtained from the literature and include annual treatment costs, as well as costs of future complications. For an arterial embolic complication we used costs of a stroke.

Indirect healthcare costs, also referred to as healthcare costs unrelated to CQF in gained life years, were estimated by using the remaining life-expectancy at the age of death (Appendix Table 8) and age-specific healthcare costs from a specifically developed tool labeled Practical Application to Include Disease Costs (PAID) (*38*). This tool distinguishes healthcare costs incurred in the last year of life and costs incurred in other years by sex, age and healthcare provider. To avoid a possible double count of influenza-related costs, we excluded healthcare costs of the disease category heart failure and diseases of arteries. We included costs of all healthcare providers available in the tool, and the weighted average of men and women was estimated using age-specific sex distributions of the Dutch population. The total indirect healthcare costs in the remaining life years was estimated using lifetables, attributing the cost incurred in a final life year to a person that died in the lifetable and cost incurred in other years to a person that survives in the lifetable. As the inclusion of indirect healthcare costs is specific for the Dutch guideline, we present results without the inclusion of indirect medical costs in the sensitivity analysis.

Direct non-medical costs include travel costs to the medical doctor, hospital, and pharmacy. We assumed that blood collection for screening was conducted at the medical doctor. Average distances to the different healthcare facilities and travel costs per kilometer were obtained from the Dutch guideline for economic evaluations in healthcare.

Indirect non-medical costs included productivity losses due to work absence were counted for screening, clinical evaluation, and complications. The duration of absence was adjusted for age-specific labor participation rates and age-specific working hours per week from Statistics Netherlands of 2016 (*39)*. The duration of absence was assumed to be half an hour for blood collection and 1.5 day for clinical evaluation. Given the seriousness of CQF-related complications, we assumed permanent work absence after developing a symptomatic aneurysm, heart failure, or arterial embolic complication. In accordance with the Dutch guideline on economic evaluations in healthcare, we used the friction approach. This method assumes that

work absence is limited to a certain friction period, as an unemployed person has replaced the deceased person after this period. We used a friction period of 85 days (*40*). Productivity loss per absent working hour was €35.07 (*40*).

Appendix Table 9. Costs in 2016 euros

Cost unit	Input	SD†	Distribution†	Scenario	Source and additional details
Direct healthcare costs					
Selection and invitation	0			11.36	Assumption: Screening occurs during routine visits
Blood collection	10.71				Dutch cost-effectiveness guideline, 2016 (*40*)
ELISA	7.00				Assumption based on (*41*)
IFA screen	9.90				List price JBH (P.M. Schneeberger, pers comm.)
IFA titration	19.80				List price JBH (P.M. Schneeberger, pers comm.)
Initial diagnostic procedure after positive IFA	1,299				Blood collection, IFA titration, PCR, CRP/standard blood tests, PET scan, TTE (all once); TEE (half of the patients); specialist consultations (3 times) (C.P. Bleeker-Rovers, pers. comm.)
Surgery	14,717			30,000	Based on 76% vascular surgeries, 19% heart valve surgeries, and 5% other kind of surgeries (S.E. van Roeden, pers. comm.) with average cost of 10,639 (*36*), 16,124 (*37*), and 8,803 (*36*).
Antibiotic treatment, per year					
First year	343				Based on treatment with doxycycline (1 dd 200 mg) and hydroxychloroquine (3 dd 200 mg) (*42*), pharmacy dispensing fee (6 times, at the assumption of delivery per 2 mo) and additional fee for first delivery (2 times), serologic antibiotic level determination (2 times) (C.P. Bleeker-Rovers, pers. comm.)
Consecutive years	297				Doxycycline and hydroxychloroquine, pharmacy dispensing fee (see first year)
Costs routine visits during treatment, per year	1,440				PCR, IFA, specialist visit, CRP/standard blood tests (all 4 times per year). A PET scan in the first year for vascular infections (C.P. Bleeker-Rovers, pers. comm.)
Follow-up					
Year 1	912				PCR, IFA, specialist visit, CRP/standard blood tests (4 times per year) (C.P. Bleeker-Rovers, pers. comm.)
Year 2	864				PCR, IFA, specialist visit, CRP/standard blood tests (3 times per year) (C.P. Bleeker-Rovers, pers. comm.)
Year 3	456				PCR, IFA, specialist visit, CRP/standard blood tests (2 times per year) (C.P. Bleeker-Rovers, pers. comm.)
Year 4 and after	228				PCR, IFA, specialist visit, CRP/standard blood tests (1 time per year) (C.P. Bleeker-Rovers, pers. comm.)
Complications, per year					
Heart failure	3,176				Van Giessen, 2016 (*43*)
Vascular prosthesis or aneurysm	2,430	358	Gamma		Prinssen, 2007 (*44*)
Embolic complication					
Year 1	12,352	1897	Gamma		Van Eeden, 2015 (*45*)
Year 2 and after	4,997	2038	Gamma		Van Eeden, 2015 (*45*), costs of the second half of the year extrapolated to a year
Other complications	0				Assumption
Indirect healthcare costs, lifelong				Excluded	
Proven CQF	60,301				PAID toolkit (*38*), based on the difference between life expectancy of CQF-related death and non-CQF-related death. Costs of heart
Probable CQF	47,183				

Cost unit	Input	SD†	Distribution†	Scenario	Source and additional details
					failure and vascular infections were excluded, because these costs could be related to CQF.
Direct non-healthcare costs					
Screening travel cost	0.42				Assumption travel costs to hospital
Initial diagnosis travel cost	11.42				Travel costs to hospital, including parking fee (2 times) (*40*)
Surgery travel cost	11.42				Travel costs to hospital, including parking fee (2 times) (*40*)
Antibiotics travel cost, per year	2.99				Travel costs to pharmacy (2 times) (*40*)
Travel cost of routine visits during treatment or follow-up	5.71				Travel costs to hospital, including parking fee (*40*)
Indirect non-healthcare costs					
Productivity loss screening	4.36–12.57				Half an hour of productivity loss (Assumption). Cost depends on age due to differences in net labor participation rates and average working hours per week.
Productivity loss initial diagnostics	105–302				1.5 d of lost productivity (Assumption). Cost depends on age due to differences in net labor participation rates and average working hours per week.
Productivity costs complication	5,936–17,089				We assumed that a CQF complication was leading to long-term work absence. Given that the friction method is the recommended approach in the Netherlands to value productivity losses, we limited the work absence of a complication to a standardized friction period of 85 d (*40*). Cost depends on age due to differences in net labor participation rates and average working hours per week.

*CQF, chronic Q fever; CRP, C-reactive protein; IFA, immunofluorescence assay; JBH, Jeroen Bosch hospital; PAID, Practical Application to Include future disease costs; PET, positron emission tomography; TEE, transesophageal echocardiography; TTE, transthoracal echocardiography.
†Used for the multivariate probabilistic sensitivity analysis.

Supplemental Results

Appendix Table 10. Subdivision of the Dutch 2017 adult population (N = 13,678,496) to Q fever incidence area using 4-digit postal codes and 3-digit postal codes

Incidence area	4-digit postal codes, no. (%)	3-digit postal codes, no. (%)
High	1,650,873 (12.07)	2,135,169 (15.61)
Middle	2,637,196 (19.28)	3,637,843 (26.60)
Low	9,390,427 (68.65)	7,905,484 (57.79)

Appendix Table 11. Subdivision of the Dutch 2017 adult population (N = 13,678,496) to specific risk groups

Population	Size (%)
Persons with diagnosed risk factor	908,248 (6.64)
Cardiovascular risk factor	462,512 (3.38)
Heart valve disorder or –prosthesis	329,112 (2.41)
Aortic aneurysm or vascular prosthesis	77,323 (0.57)
Congenital heart anomaly	40,968 (0.30)
Endocarditis	15,109 (0.11)
Immunocompromised status	445,736 (3.26)
Underlying disease*	158,858 (1.16)
Medication use	286,878 (2.10)
Rheumatoid arthritis	217,764 (1.59)
Inflammatory bowel disease	69,115 (0.51)
Persons without diagnosed risk factor	12,770,248 (93.36)
Age ≥60 y	3,633,184 (26.56)
Undiagnosed cardiovascular risk factor	141,221 (1.03)
Heart valve disorder	96,311 (0.70)

Population	Size (%)
Aortic aneurysm	44,911 (0.33)
No risk factor†	3,491,963 (25.53)
Age 18–59 y	9,137,064 (66.80)
Undiagnosed cardiovascular risk factor	2,379 (0.02)
Heart valve disorder	– (0.00)
Aortic aneurysm	2,379 (0.02)
No risk factor†	9,134,685 (66.78)

*Includes HIV infection, asplenia, spleen dysfunction, malignancy (e.g., leukemia) or bone marrow transplant.

†No risk factor is defined here as patients without a cardiovascular risk factor or compromised immune system.

Appendix Table 12. Estimation of the prevalence and number of *Coxiella burnetii*–infected individuals and CQF patients*

Screening population	Prevalence scenario	Population size	Seroprevalence	*C. burnetii* infections	CQF prevalence after epidemic	CQF patients after epidemic	CQF prevalence at screening	CQF patients at screening	Proven CQF patients at screening	Probable CQF patients at screening	Possible CQF patients at screening
High incidence area											
CVRF patients	Low	55,821	215	1,202	26.2	146	6.4	36	25	11	–
	High	72,197	1,070	7,725	130.1	939	62.4	451	311	140	–
IC patients	Low	53,796	215	1,159	14.8	80	3.6	20	13	6	–
	High	69,578	1,070	7,445	73.4	511	35.2	245	169	76	–
Age ≥60 y, unknown RF	Low	438,493	215	9,444	1.7	74	0.4	18	10	4	4
	High	567,128	1,070	60,683	8.4	477	4.0	229	119	54	56
Age 18–59 y, unknown RF	Low	1,102,763	215	23,750	0.4	49	0.1	12	0	0	12
	High	1,426,266	1,070	152,610	2.2	317	1.1	152	4	2	146
Middle incidence area											
CVRF patients	Low	89,172	15	135	1.8	16	0.5	4	3	1	–
	High	123,007	230	2,829	28.0	344	13.4	165	114	51	–
IC patients	Low	85,937	15	131	1.0	9	0.3	2	2	1	–
	High	118,545	230	2,727	15.8	187	7.6	90	62	28	–
Age ≥60 y, unknown RF	Low	700,473	15	1,064	0.1	8	0.0	2	1	0	1
	High	966,258	230	22,224	1.8	175	0.9	84	44	20	21
Age 18–59 y, unknown RF	Low	1,761,614	15	2,677	0.0	6	0.0	1	0	0	1
	High	2,430,034	230	55,891	0.5	116	0.2	56	1	1	54
Low incidence area											
CVRF patients	Low	317,519	2.7	87	0.3	11	0.1	3	2	1	–
	High	267,308	100	2,673	12.2	325	5.8	156	108	48	–
IC patients	Low	306,002	2.7	84	0.2	6	0.0	1	1	0	–
	High	257,613	100	2,576	6.9	177	3.3	85	58	26	–
Age ≥60 y, unknown RF	Low	2,494,218	2.7	685	0.0	5	0.0	1	1	0	0
	High	2,099,798	100	20,998	0.8	165	0.4	79	41	19	19
Age 18–59 y, unknown RF	Low	6,272,687	2.7	1,724	0.0	4	0.0	1	0	0	1
	High	5,280,764	100	52,808	0.2	110	0.1	53	1	1	51
Total	Low	13,678,496	31	42,143	0.3	414	0.1	102	57	26	19
	High	13,678,496	286	391,188	2.8	3,842	1.3	1,844	1,032	465	347

*CQF, chronic Q fever; CVRF, cardiovascular risk factor; IC, immunocompromised; RF, risk factor.

Appendix Table 13. Screening outcomes at a screening participation rate of 50%*

Screening population	Prevalence scenario	Persons screened	ELISA positive	IFA positive	CQF patients detected	NNS CQF	Proven CQF patients detected	NNS proven CQF
High incidence area								
CVRF patients	Low	27,911	856	28	18	1,552	12	2,252
	High	36,098	2,689	288	225	160	155	232
IC patients	Low	26,898	821	20	10	2,750	7	3,990
	High	34,789	2,544	184	123	284	85	412
Age >60 y, unknown RF	Low	219,247	6,656	21	9	24,020	5	46,141
	High	283,564	20,292	190	86	3,281	60	4,760
Age 18–59 y, unknown RF	Low	551,381	16,731	29	6	90,913	0	3,585,959
	High	713,133	50,927	225	3	254,977	2	369,966
Middle incidence area								
CVRF patients	Low	44,586	925	3	2	22,002	1	31,924
	High	61,503	1,950	105	83	745	57	1,081
IC patients	Low	42,969	891	2	1	38,980	1	56,559
	High	59,273	1,862	67	45	1,320	31	1,915
Age >60 y, unknown RF	Low	350,237	7,261	2	1	340,477	1	654,042
	High	483,129	15,017	70	32	15,263	22	22,146
Age 18–59 y, unknown RF	Low	880,807	18,259	3	1	1,288,685	0	50,830,867
	High	1,215,017	37,728	82	1	1,186,195	1	1,721,146
Low incidence area								
CVRF patients	Low	158,759	3,197	2	1	121,642	1	176,499
	High	133,654	3,354	100	78	1,713	54	2,486
IC patients	Low	153,001	3,081	1	1	215,509	0	312,699
	High	128,807	3,216	64	42	3,036	29	4,405
Age >60 y, unknown RF	Low	1,247,109	25,107	2	1	1,882,392	0	3,615,996
	High	1,049,899	26,057	66	30	35,104	21	50,936
Age 18–59 y, unknown RF	Low	3,136,344	63,141	2	0	7,124,742	0	281,028,271
	High	2,640,382	65,495	78	1	2,728,249	1	3,958,636

*CQF, chronic Q fever; CVRF, cardiovascular risk factor; IC, immunocompromised; NNS, number needed to screen; RF, risk factor.

Appendix Table 14. Clinical and health impact of the analyzed screening strategies as compared to no screening at a screening participation rate of 50%*

Screening population	Prevalence scenario	Additional antibiotic courses	Complications averted	Surgeries averted	CQF-related deaths averted	Life years saved	QALYs gained
High incidence area							
CVRF patients	Low	4.1	−8.4	−4.3	−2.1	15.2	17.1
	High	51.5	−104.7	−53.9	−25.8	190.2	214.9
IC patients	Low	2.2	−4.5	−2.3	−1.1	8.3	9.3
	High	28.0	−56.9	−29.3	−14.0	103.4	116.9
Age >60 y, unknown RF	Low	1.6	−3.2	−1.6	−0.8	5.8	6.6
	High	19.8	−40.1	−20.7	−9.9	72.9	82.4
Age 18–59 y, unknown RF	Low	0.1	−0.1	−0.1	−0.0	0.2	0.2
	High	0.6	−1.3	−0.7	−0.3	2.4	2.7
Middle incidence area							
CVRF patients	Low	0.5	−0.9	−0.5	−0.2	1.7	1.9
	High	18.9	−38.3	−19.7	−9.4	69.6	78.7
IC patients	Low	0.3	−0.5	−0.3	−0.1	0.9	1.1
	High	10.3	−20.9	−10.7	−5.1	37.9	42.8
Age >60 y, unknown RF	Low	0.2	−0.4	−0.2	−0.1	0.7	0.7
	High	7.2	−14.7	−7.6	−3.6	26.7	30.2
Age 18–59 y, unknown RF	Low	0.0	−0.0	−0.0	−0.0	0.0	0.0
	High	0.2	−0.5	−0.2	−0.1	0.9	1.0
Low incidence area							
CVRF patients	Low	0.3	−0.6	−0.3	−0.1	1.1	1.2
	High	17.8	−36.2	−18.7	−8.9	65.8	74.4
IC patients	Low	0.2	−0.3	−0.2	−0.1	0.6	0.7
	High	9.7	−19.7	−10.1	−4.9	35.8	40.5
Age >60 y, unknown RF	Low	0.1	−0.2	−0.1	−0.1	0.4	0.5
	High	6.8	−13.9	−7.2	−3.4	25.2	28.5
Age 18–59 y, unknown RF	Low	0.0	−0.0	−0.0	−0.0	0.0	0.0
	High	0.2	−0.4	−0.2	−0.1	0.8	0.9

*CQF, chronic Q fever; CVRF, cardiovascular risk factor; IC, immunocompromised; QALY, quality-adjusted life year;, RF, risk factor.

Appendix Table 15. Incremental costs of the analyzed screening strategies as compared to no screening at a screening participation rate of 50%*

Screening population	Prevalence scenario	Screening costs, €	Direct HC costs, €	Non-HC costs, direct and indirect, €	Total societal costs, excluding indirect HC costs, €	Indirect HC costs, €	Total societal costs (including indirect HC costs, €
High incidence area							
CVRF patients	Low	503,270	−144,557	81,542	440,256	103,818	544,074
	High	671,548	−1,892,276	−155,227	−1,375,955	1,301,471	−74,484
IC patients	Low	484,832	−73,132	148,657	560,358	56,473	616,831
	High	644,881	−993,980	−88,602	−437,702	707,956	270,255
Age ≥60 y, unknown RF	Low	3,948,773	−52,244	527,743	4,424,273	39,806	4,464,079
	High	5,226,068	−679,387	657,153	5,203,834	499,016	5,702,850
Age 18–59 y, unknown RF	Low	9,930,185	9,116	6,290,432	16,229,733	1,288	16,231,021
	High	13,136,941	113,863	8,142,174	21,392,977	16,148	21,409,125
Middle incidence area							
CVRF patients	Low	798,757	−16,291	163,934	946,400	11,700	958,100
	High	1,110,506	−693,011	123,447	540,942	476,640	1,017,582
IC patients	Low	769,765	−8,242	273,324	1,034,847	6,364	1,041,211
	High	1,069,382	−364,027	266,486	971,841	259,276	1,231,117
Age ≥60 y, unknown RF	Low	6,273,987	−5,888	846,345	7,114,444	4,486	7,118,930
	High	8,705,394	−248,813	1,157,466	9,614,047	182,756	9,796,803
Age 18–59 y, unknown RF	Low	15,778,334	1,027	10,047,828	25,827,189	145	25,827,334
	High	21,890,903	41,700	13,862,862	35,795,465	5,914	35,801,379
Low incidence area							
CVRF patients	Low	2,843,033	−10,492	591,186	3,423,727	7,535	3,431,262
	High	2,401,947	−654,782	398,729	2,145,894	450,347	2,596,241
IC patients	Low	2,739,902	−5,308	981,177	3,715,771	4,099	3,719,870
	High	2,314,029	−343,946	719,500	2,689,583	244,973	2,934,557
Age ≥60 y, unknown RF	Low	22,332,648	−3,792	3,014,362	25,343,218	2,889	25,346,107
	High	18,851,093	−235,088	2,528,039	21,144,045	172,674	21,316,719
Age 18–59 y, unknown RF	Low	56,164,141	662	35,777,732	91,942,535	93	91,942,628
	High	47,406,356	39,400	30,122,503	77,568,258	5,588	77,573,846

*CQF, chronic Q fever; CVRF, cardiovascular risk factor; HC, healthcare; IC, immunocompromised; RF, risk factor.

Appendix Table 16. Costs of screening of 50% of all adults in the Netherlands as compared to no screening at all*

Cost component	Without screening, €, million	Screening, €, million	Difference, €, million
Direct healthcare costs			
Screening	–	123.43	123.43
Blood sampling	–	73.24	73.24
ELISA	–	47.87	47.87
IFA	–	2.32	2.32
Treatment of CQF	33.43	31.84	–1.59
Diagnostic procedures	1.39	2.18	0.79
Surgeries	8.80	6.17	–2.64
Antibiotics	0.51	0.61	0.09
Follow-up during treatment	1.78	2.10	0.32
Follow-up after treatment	2.57	3.26	0.69
Complications	18.37	13.20	–5.17
Indirect healthcare costs	–	4.32	4.32
Direct non-healthcare costs	0.15	3.07	2.92
Travel costs screening	–	2.89	2.89
Travel costs treatment of CQF	0.15	0.18	0.03
Indirect non-healthcare costs	4.20	59.01	54.82
Productivity loss screening	–	55.92	55.92
Productivity loss treatment of CQF	4.20	3.09	–1.10
Total societal costs	37.77	217.35	179.58

*CQF, chronic Q-fever; IFA, immunofluorescence assay.

Appendix Table 17. Cost-effectiveness of screening strategies as compared to no screening at a screening participation rate of 50%*

Screening population	Prevalence scenario	Screening: Costs, €, million†	Screening: QALYs†	No screening: Costs, €, million†	No screening: QALYs†	Difference: Costs, €, million†	Difference: Total QALYs†	ICER, €/QALY gained
High incidence area								
CVRF patients	Low	1.44	174.9	0.89	157.8	0.54	17.1	31,737
	High	11.11	2,192.7	11.19	1,977.8	−0.07	214.9	Cost-saving
IC patients	Low	1.15	95.1	0.53	85.8	0.62	9.3	66,145
	High	6.94	1,192.8	6.67	1,075.9	0.27	116.9	2,312
Age ≥60 y, unknown RF	Low	4.78	165.5	0.32	158.9	4.46	6.6	679,136
	High	9.70	2,074.8	4.00	1,992.4	5.70	82.4	69,208
Age 18–59 y, unknown RF	Low	16.25	259.7	0.02	259.5	16.23	0.2	76,308,665
	High	21.62	3,255.4	0.21	3,252.8	21.41	2.7	8,029,064
Middle incidence area								
CVRF patients	Low	1.06	19.7	0.10	17.8	0.96	1.9	495,918
	High	5.11	803.0	4.10	724.3	1.02	78.7	12,929
IC patients	Low	1.10	10.7	0.06	9.7	1.04	1.1	990,755
	High	3.67	436.8	2.44	394.0	1.23	42.8	28,755
Age ≥60 y, unknown RF	Low	7.15	18.7	0.04	17.9	7.12	0.7	9,610,222
	High	11.26	759.9	1.47	729.7	9.80	30.2	324,632
Age 18–59 y, unknown RF	Low	25.83	29.3	0.00	29.2	25.83	0.0	1,077,459,984
	High	35.88	1,192.2	0.08	1,191.3	35.80	1.0	36,661,479
Low incidence area								
CVRF patients	Low	3.50	12.7	0.06	11.5	3.43	1.2	2,757,608
	High	6.47	758.7	3.87	684.4	2.60	74.4	34,912
IC patients	Low	3.76	6.9	0.04	6.2	3.72	0.7	5,495,846
	High	5.24	412.7	2.31	372.3	2.93	40.5	72,544
Age ≥60 y, unknown RF	Low	25.37	12.0	0.02	11.5	25.35	0.5	53,126,291
	High	22.70	717.9	1.38	689.4	21.32	28.5	747,603
Age 18–59 y, unknown RF	Low	91.94	18.8	0.00	18.8	91.94	0.0	5,955,497,518
	High	77.65	1,126.5	0.07	1,125.6	77.57	0.9	84,075,394

*CQF, chronic Q-fever; CVRF, cardiovascular risk factor; IC, immunocompromised; ICER, incremental cost-effectiveness ratio; QALY, quality-adjusted life year; RF, risk factor.
†In CQF patients only, except costs of screening.

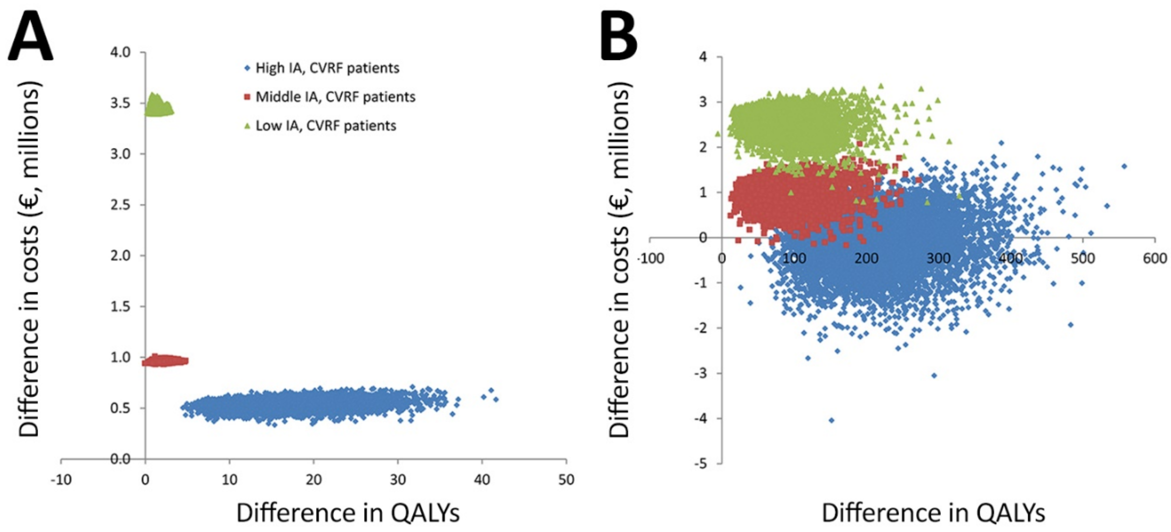

Appendix Figure 2. Results of the multivariate sensitivity analysis using 10,000 simulations for screening of patients with a cardiovascular risk factor in high, middle, and low incidence areas for the (A) low CQF prevalence scenario and (B) high CQF prevalence scenario. CQF, chronic Q fever; CVRF, cardiovascular risk factor; IA, incidence area; QALY, quality-adjusted life year.

Appendix Table 18. Cost-effectiveness of screening without the inclusion of indirect medical costs

Screening population	CQF prevalence scenario	Difference in QALYs	Difference in costs, without indirect HC costs	ICER, €/QALY gained, without indirect HC costs
High incidence area				
CVRF patients	Low	17.1	440,256	25,681
	High	214.9	–1,375,955	–6,402
IC patients	Low	9.3	560,358	60,090
	High	116.9	–437,702	–3,744
Age ≥60 y, unknown RF	Low	6.6	4,424,273	673,080
	High	82.4	5,203,834	63,152
Age 18–59 y, unknown RF	Low	0.2	16,229,733	76,302,609
	High	2.7	21,392,977	8,023,009
Middle incidence area				
CVRF patients	Low	1.9	946,400	489,862
	High	78.7	540,942	6,873
IC patients	Low	1.1	1,034,847	984,699
	High	42.8	971,841	22,699
Age ≥60 y, unknown RF	Low	0.7	7,114,444	9,604,166
	High	30.2	9,614,047	318,576
18–59 y, unknown RF	Low	0.0	25,827,189	1,077,453,928
	High	1.0	35,795,465	36,655,423
Low incidence area				
CVRF patients	Low	1.2	3,423,727	2,751,552
	High	74.4	2,145,894	28,856
IC patients	Low	0.7	3,715,771	5,489,790
	High	40.5	2,689,583	66,488
Age ≥ 60 y, unknown RF	Low	0.5	25,343,218	53,120,236
	High	28.5	21,144,045	741,547
Age 18–59 y, unknown RF	Low	0.0	91,942,535	5,955,491,462
	High	0.9	77,568,258	84,069,338

*CQF, chronic Q-fever; CVRF, cardiovascular risk factor; HC, healthcare; IC, immunocompromised; ICER, incremental cost-effectiveness ratio; QALY, quality-adjusted life year; RF, risk factor.

References

1. Vermeer-de Bondt PE, Schoffelen T, Vanrolleghem AM, Isken LD, van Deuren M, Sturkenboom MC, et al. Coverage of the 2011 Q fever vaccination campaign in the Netherlands, using retrospective population-based prevalence estimation of cardiovascular risk-conditions for chronic Q fever. PLoS One. 2015;10:e0123570. PubMed https://doi.org/10.1371/journal.pone.0123570

2. d'Arcy JL, Coffey S, Loudon MA, Kennedy A, Pearson-Stuttard J, Birks J, et al. Large-scale community echocardiographic screening reveals a major burden of undiagnosed valvular heart disease in older people: the OxVALVE Population Cohort Study. Eur Heart J. 2016;37:3515–22. PubMed https://doi.org/10.1093/eurheartj/ehw229

3. Pleumeekers HJ, Hoes AW, van der Does E, van Urk H, Hofman A, de Jong PT, et al. Aneurysms of the abdominal aorta in older adults. The Rotterdam Study. Am J Epidemiol. 1995;142:1291–9. PubMed https://doi.org/10.1093/oxfordjournals.aje.a117596

4. van Hoek AJ, Andrews N, Waight PA, Stowe J, Gates P, George R, et al. The effect of underlying clinical conditions on the risk of developing invasive pneumococcal disease in England. J Infect. 2012;65:17–24. PubMed https://doi.org/10.1016/j.jinf.2012.02.017

5. Volksgezondheidenzorg.info. Rheumatoid arthritis (RA) > numbers & context > current situation [In Dutch]. 2017 [cited 2017 May 1]. https://www.volksgezondheidenzorg.info/onderwerp/reumato%C3%AFde-artritis-ra/cijfers-context/huidige-situatie#node-prevalentie-van-reumatoïde-artritis

6. de Groof EJ, Rossen NG, van Rhijn BD, Karregat EP, Boonstra K, Hageman I, et al. Burden of disease and increasing prevalence of inflammatory bowel disease in a population-based cohort in the Netherlands. Eur J Gastroenterol Hepatol. 2016;28:1065–72. PubMed https://doi.org/10.1097/MEG.0000000000000660

7. Eldin C, Mélenotte C, Mediannikov O, Ghigo E, Million M, Edouard S, et al. From Q fever to *Coxiella burnetii* infection: a paradigm change. Clin Microbiol Rev. 2017;30:115–90. PubMed https://doi.org/10.1128/CMR.00045-16

8. Wegdam-Blans MC, Kampschreur LM, Nabuurs-Franssen MH, Renders NHM, Delsing CE, Bijlmer HA. Nederlandse consensus chronische Q-koorts. Tijdschrift voor Infectieziekten 2011;6:71–3.

9. Li JS, Sexton DJ, Mick N, Nettles R, Fowler VG Jr, Ryan T, et al. Proposed modifications to the Duke criteria for the diagnosis of infective endocarditis. Clin Infect Dis. 2000;30:633–8. PubMed https://doi.org/10.1086/313753

10. van der Hoek W, Hogema BM, Dijkstra F, Rietveld A, Wijkmans CJ, Schneeberger PM, et al. Relation between Q fever notifications and *Coxiella burnetii* infections during the 2009 outbreak in the Netherlands. Euro Surveill. 2012;17:20058. PubMed

11. Kampschreur LM, Oosterheert JJ, Hoepelman AI, Lestrade PJ, Renders NH, Elsman P, et al. Prevalence of chronic Q fever in patients with a history of cardiac valve surgery in an area where *Coxiella burnetii* is epidemic. Clin Vaccine Immunol. 2012;19:1165–9. PubMed https://doi.org/10.1128/CVI.00185-12

12. Wegdam-Blans MC, Stokmans RA, Tjhie JH, Korbeeck JM, Koopmans MP, Evers SM, et al. Targeted screening as a tool for the early detection of chronic Q fever patients after a large outbreak. Eur J Clin Microbiol Infect Dis. 2013;32:353–9. PubMed https://doi.org/10.1007/s10096-012-1749-9

13. Hagenaars JC, Wever PC, van Petersen AS, Lestrade PJ, de Jager-Leclercq MG, Hermans MH, et al. Estimated prevalence of chronic Q fever among *Coxiella burnetii* seropositive patients with an abdominal aortic/iliac aneurysm or aorto-iliac reconstruction after a large Dutch Q fever outbreak. J Infect. 2014;69:154–60. PubMed https://doi.org/10.1016/j.jinf.2014.03.009

14. Schoffelen T, Kampschreur LM, van Roeden SE, Wever PC, den Broeder AA, Nabuurs-Franssen MH, et al. *Coxiella burnetii* infection (Q fever) in rheumatoid arthritis patients with and without anti-TNFα therapy. Ann Rheum Dis. 2014;73:1436–8. PubMed https://doi.org/10.1136/annrheumdis-2014-205455

15. Wegdam-Blans MC, Kampschreur LM, Delsing CE, Bleeker-Rovers CP, Sprong T, van Kasteren ME, et al.; Dutch Q fever Consensus Group. Chronic Q fever: review of the literature and a proposal of new diagnostic criteria. J Infect. 2012;64:247–59. PubMed https://doi.org/10.1016/j.jinf.2011.12.014

16. Morroy G, van der Hoek W, Albers J, Coutinho RA, Bleeker-Rovers CP, Schneeberger PM. Population screening for chronic Q-fever seven years after a major outbreak. PLoS One. 2015;10:e0131777. PubMed https://doi.org/10.1371/journal.pone.0131777

17. Pijnacker R, Reimerink J, Smit LAM, van Gageldonk-Lafeber AB, Zock JP, Borlée F, et al. Remarkable spatial variation in the seroprevalence of *Coxiella burnetii* after a large Q fever epidemic. BMC Infect Dis. 2017;17:725. PubMed https://doi.org/10.1186/s12879-017-2813-y

18. Brandwagt DA, Herremans T, Schneeberger PM, Hackert VH, Hoebe CJ, Paget J, et al. Waning population immunity prior to a large Q fever epidemic in the south of the Netherlands. Epidemiol Infect. 2016;144:2866–72. PubMed https://doi.org/10.1017/S0950268816000741

19. de Lange MMA, Scheepmaker A, van der Hoek W, Leclercq M, Schneeberger PM. Risk of chronic Q fever in patients with cardiac valvulopathy, seven years after a large epidemic in the Netherlands. PLoS One. 2019;14:e0221247. PubMed https://doi.org/10.1371/journal.pone.0221247

20. Buijs SB, Oosterheert JJ, Van Roeden SE, Kampschreur LM, Hoepelman AI, Wever PC, et al. Still new chronic Q fever cases diagnosed more than five years after a large Q fever outbreak [cited 2019 Sep 1]. https://www.escmid.org/escmid_publications/escmid_elibrary/material/?mid=67200

21. Wielders CC, van Loenhout JA, Morroy G, Rietveld A, Notermans DW, Wever PC, et al. Long-term serological follow-up of acute Q-fever patients after a large epidemic. PLoS One. 2015;10:e0131848. PubMed https://doi.org/10.1371/journal.pone.0131848

22. Frosinski J, Hermann B, Maier K, Boden K. Enzyme-linked immunosorbent assays in seroprevalence studies of Q fever: the need for cut-off adaptation and the consequences for prevalence data. Epidemiol Infect. 2016;144:1148–52. PubMed https://doi.org/10.1017/S0950268815002447

23. Kampschreur LM, Hagenaars JC, Wielders CC, Elsman P, Lestrade PJ, Koning OH, et al. Screening for *Coxiella burnetii* seroprevalence in chronic Q fever high-risk groups reveals the magnitude of the Dutch Q fever outbreak. Epidemiol Infect. 2013;141:847–51. PubMed https://doi.org/10.1017/S0950268812001203

24. Million M, Thuny F, Richet H, Raoult D. Long-term outcome of Q fever endocarditis: a 26-year personal survey. Lancet Infect Dis. 2010;10:527–35. PubMed https://doi.org/10.1016/S1473-3099(10)70135-3

25. van Roeden SE, Wever PC, Kampschreur LM, Gruteke P, van der Hoek W, Hoepelman AIM, et al. Chronic Q fever–related complications and mortality: data from a nationwide cohort. Clin Microbiol Infect. 2019;25:1390–8. PubMed

26. M Versteegh M, M Vermeulen K, M A A Evers S, de Wit GA, Prenger R, A Stolk E. Dutch tariff for the five-level version of EQ-5D. Value Health. 2016;19:343–52. PubMed https://doi.org/10.1016/j.jval.2016.01.003

27. Franklin M, Wailoo A, Dayer MJ, Jones S, Prendergast B, Baddour LM, et al. The cost-effectiveness of antibiotic prophylaxis for patients at risk of infective endocarditis. Circulation. 2016;134:1568–78. PubMed https://doi.org/10.1161/CIRCULATIONAHA.116.022047

28. Timmers TK, van Herwaarden JA, de Borst GJ, Moll FL, Leenen LP. Long-term survival and quality of life after open abdominal aortic aneurysm repair. World J Surg. 2013;37:2957–64. PubMed https://doi.org/10.1007/s00268-013-2206-3

29. Calvert MJ, Freemantle N, Cleland JG. The impact of chronic heart failure on health-related quality of life data acquired in the baseline phase of the CARE-HF study. Eur J Heart Fail. 2005;7:243–51. PubMed https://doi.org/10.1016/j.ejheart.2005.01.012

30. Stouthard ME, Essink-Bot ML, Bonsel GJ, Barendregt JJM, Kramers PGN, van de Water HPA, et al. Disability weights for diseases in the Netherlands [cited 2019 Sep 1]. https://pure.uva.nl/ws/files/3238153/3276_ddw.pdf

31. World Health Organization. Global burden of disease 2004 update: disability weights for diseases and conditions. Geneva: The Organization; 2004.

32. Statistics Netherlands. Life-expectancy; gender, age (per year and period of five years) [cited 2017 Dec 1]. http://statline.cbs.nl/Statweb/publication/?DM=SLNL&PA=37360ned&D1=0&D2=0&D3=a&D4=94-95%2cl&HDR=G1%2cT&STB=G2%2cG3&VW=T

33. van Geldorp MW, Eric Jamieson WR, Kappetein AP, Ye J, Fradet GJ, Eijkemans MJ, et al. Patient outcome after aortic valve replacement with a mechanical or biological prosthesis: weighing lifetime anticoagulant-related event risk against reoperation risk. J Thorac Cardiovasc Surg. 2009;137:881–6, 886e1–5.

34. van Roeden SE, Bleeker-Rovers CP, de Regt MJA, Kampschreur LM, Hoepelman AIM, Wever PC, et al. Treatment of chronic Q fever: clinical efficacy and toxicity of antibiotic regimens. Clin Infect Dis. 2018;66:719–26. PubMed https://doi.org/10.1093/cid/cix886

35. Statistics Netherlands. Consumer prices; price index 2015=100 [cited 2017 Dec 1]. http://statline.cbs.nl/Statweb/publication/?DM=SLEN&PA=83131ENG&D1=0&D2=0&D3=64,77,90,103,116,129,142,155,168,181,194,207,220,233,246,259,272,285&LA=EN&HDR=T&STB=G1,G2&VW=T

36. Mangen MJ, Rozenbaum MH, Huijts SM, van Werkhoven CH, Postma DF, Atwood M, et al. Cost-effectiveness of adult pneumococcal conjugate vaccination in the Netherlands. Eur Respir J. 2015;46:1407–16. PubMed https://doi.org/10.1183/13993003.00325-2015

37. Burgers LT, Vahl AC, Severens JL, Wiersema AM, Cuypers PW, Verhagen HJ, et al. Cost-effectiveness of elective endovascular aneurysm repair versus open surgical repair of abdominal

aortic aneurysms. Eur J Vasc Endovasc Surg. 2016;52:29–40. PubMed https://doi.org/10.1016/j.ejvs.2016.03.001

38. van Baal PH, Wong A, Slobbe LC, Polder JJ, Brouwer WB, de Wit GA. Standardizing the inclusion of indirect medical costs in economic evaluations. Pharmacoeconomics. 2011;29:175–87. PubMed https://doi.org/10.2165/11586130-000000000-00000

39. Statistics Netherlands. Labour participation; key figures [cited 2017 Dec 1]. http://statline.cbs.nl/Statweb/publication/?DM=SLNL&PA=82309NED&D1=22-23&D2=a&D3=18-22&D4=0&D5=69&HDR=G1,T&STB=G2,G3,G4&VW=T

40. National Health Care Institute. Guideline for economic evaluations in healthcare [cited 2017 Dec 1]. https://english.zorginstituutnederland.nl/publications/reports/2016/06/16/guideline-for-economic-evaluations-in-healthcare

41. van der Hoek W, Wielders CC, Schimmer B, Wegdam-Blans MC, Meekelenkamp J, Zaaijer HL, et al. Detection of phase I IgG antibodies to *Coxiella burnetii* with EIA as a screening test for blood donations. Eur J Clin Microbiol Infect Dis. 2012;31:3207–9. PubMed https://doi.org/10.1007/s10096-012-1686-7

42. National Institute for Public Health and the Environment. LCI-guideline Q-fever [in Dutch]. 2014 [cited 2017 May 24]. https://lci.rivm.nl/richtlijnen/q-koorts

43. van Giessen A, Boonman-de Winter LJ, Rutten FH, Cramer MJ, Landman MJ, Liem AH, et al. Cost-effectiveness of screening strategies to detect heart failure in patients with type 2 diabetes. Cardiovasc Diabetol. 2016;15:48. PubMed https://doi.org/10.1186/s12933-016-0363-z

44. Prinssen M, Buskens E, de Jong SE, Buth J, Mackaay AJ, van Sambeek MR, et al.; DREAM trial participants. Cost-effectiveness of conventional and endovascular repair of abdominal aortic aneurysms: results of a randomized trial. J Vasc Surg. 2007;46:883–90. Erratum in: J Vasc Surg. 2008;47:483. PubMed https://doi.org/10.1016/j.jvs.2007.07.033

45. van Eeden M, van Heugten C, van Mastrigt GA, van Mierlo M, Visser-Meily JM, Evers SM. The burden of stroke in the Netherlands: estimating quality of life and costs for 1 year poststroke. BMJ Open. 2015;5:e008220. PubMed https://doi.org/10.1136/bmjopen-2015-008220
